# Computational Insights into the Energetics of Single C_2_–C_10_ Aliphatic Moieties Adsorbed on the Hydrogenated Silicon (111) Surface

**DOI:** 10.1021/acs.langmuir.4c05103

**Published:** 2025-04-08

**Authors:** Francesco Buonocore, Sara Marchio, Simone Giusepponi, Massimo Celino

**Affiliations:** Italian National Agency for New Technologies, Energy and Sustainable Economic Development (ENEA)−C. R. Casaccia, Via Anguillarese 301, 00123 Rome, Italy

## Abstract

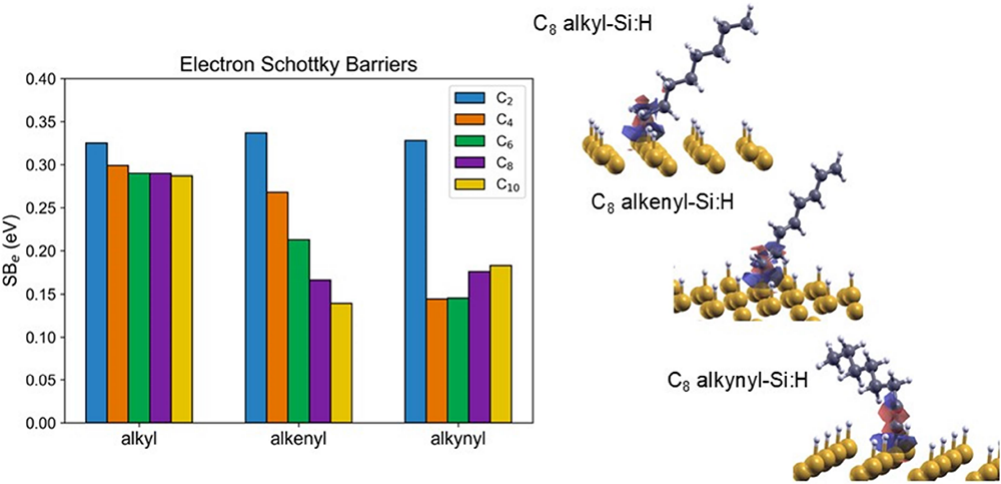

Silicon’s versatility as a semiconductor renders it indispensable across various domains, including electronics, sensors, and photovoltaics. Modifying hydrogen-terminated silicon surfaces with moiety adsorption offers a method to tailor the material’s properties for specific applications. In this study, we employ ab initio density functional theory calculations to explore the energetics of single alkyl, 1-alkenyl, and 1-alkynyl moieties chemisorbed on the hydrogen-terminated silicon (111) surface. We analyze the interfacial dipole induced by Si–C bond formation that determines the Schottky barrier and examine the alignment of the frontier orbital energy levels with the silicon band structure to investigate charge transfer based on the tunneling mechanism. Our findings provide valuable insights into how aliphatic moiety functionalization affects interfacial electronic properties, offering clues for optimizing silicon-based devices.

## 1 Introduction

Silicon, with its exceptional semiconductor properties, is a fundamental material in modern technology, underpinning a vast array of applications from integrated circuits and sensors to photovoltaic cells.^1−13^ The versatility and efficiency of silicon-based devices are significantly enhanced by surface modifications, which allow for precise tuning of electronic, optical, and chemical properties. Unfortunately, the presence of silicon oxide during the fabrication processes leads to lower quality or nonfunctioning devices. Indeed, silicon oxide constitutes an insulating layer that must be prevented when electrical contact is formed. One of the most adopted strategies to avoid oxide formation involves the functionalization of hydrogen-terminated silicon (H–Si) surfaces with molecular monolayers.^14^ Actually, there is a growing interest in developing alternative functionalization methods that avoid oxide formation, thereby preserving the conductive properties of silicon. Since the first report of Si–C alkyl monolayers,^15^ the Si–C bond has been proven to be more stable than the Si–H bond. In this context, the aliphatic chain molecules represent a convincing option to prevent the formation of undesired oxide on the Si surface, creating a direct interface that maintains desirable conductive properties. This technique enables the customization of surface characteristics to meet the specific demands of various applications. Moreover, the formation of molecular monolayers based on aliphatic chains has been proposed^16^ to improve the performance of low-cost polycrystalline semiconductors. The impact of such functionalization on the electronic structure and interfacial properties of silicon, however, requires thorough investigation.^17,18^

Short aliphatic chains are known to form more ordered monolayers than longer ones.^19,20^ However, monolayers derived from short alkenes and alkynes are more difficult to synthesize using wet chemistry. For this reason, different approaches using gaseous aliphatic molecules at elevated pressure have been proposed.^20^

Several theoretical studies have been dedicated to the functionalization of the hydrogen-terminated silicon (111) (H–Si(111)) surface by aliphatic chains. Zhang et al. investigated the optimal packing structure of C_18_ alkyl monolayers on a Si(111) surface based on molecular dynamics simulations.^21^ In this work, the aliphatic chains are generically indicated as C_N_, where *N* represents the number of C atoms. The morphology of alkyl monolayers from C_2_ to C_18_ on the H–Si(111) surface was investigated by the molecular simulation method.^22^ Numerous works have been based on ab initio density functional theory (DFT). The binding energies of 1-alkenes and 1-alkynes to the H–Si surface were calculated to determine the thermodynamic feasibility of the monolayer structures. It was demonstrated that coverage as high as 75% is possible for octadecenyl chains due to the significantly larger reaction exothermicity of 1-alkynes.^23^ The functionalization of the hydrogenated Si surface with C_18_ alkyl monolayers to form hydrophobic coatings was explored.^24^ The sequence of the stability of C_N_ alkyls with *N* ranging from 1 to 10 for different linkers was assessed.^25^ Ab initio calculations were performed to investigate the reactivity of silicon terminated with aliphatic chains toward oxidizing molecules to understand the mechanisms that avoid the oxidation of the silicon substrate.^26^ Moreover, the work function of H–Si(111) was shown to be modified over a range of 1.7 eV through the adsorption of C_N_ alkyl monolayers with *N* ranging from 1 to 14.^27^

This study utilizes ab initio DFT calculations to investigate the effects of single aliphatic chain moiety adsorption on the energetics of H–Si(111) surfaces. We calculated using DFT the minimal energy geometrical configurations of alkyl (C_N_H_2N+1_), 1-alkenyl (C_N_H_N+1_), and 1-alkynyl (C_N_H_2N-3_) moieties, with the number of carbon atoms *N* varying from 2 to 10, adsorbed on the surface. It has been reported that the functionalization with 1-alkenes and 1-alkynes of the H–Si(111) surface results in the formation of alkyl and 1-alkenyl monolayers, respectively.^28^ However, this work adopts a broader perspective to investigate the distinct effects that single, double, or triple C–C bonds near the surface in individual aliphatic moieties have on the energetics of the inorganic/organic interface. These effects are analyzed in the context of replacing one H atom attached to the Si surface with the aliphatic moiety, resulting in the substitution of one Si–H bond with a Si–C bond, and the chemisorption of the moiety. Moiety chemisorption has a substantial impact on the modification of the electronic properties of the interface, differently from physisorption, where the molecule weakly interacts via dispersion forces. By examining the charge distribution, the dipole formation, and the alignment of molecular energy levels at the Si–C interface, our research seeks to provide a detailed understanding of how these modifications influence interface properties, such as Schottky and tunneling barriers, offering valuable insights for optimizing the design and functionality of silicon-based devices. The investigation of single moiety adsorption is a prelude to further studies, where the interactions among moieties in monolayers will be explored.

## 2 Theoretical Methods

The computational approach was based on DFT and the pseudopotential plane-wave method using the PWSCF code as implemented in the QUANTUM-ESPRESSO package.^29,30^ To account for van der Waals interactions, we employed the nonlocal DFT functional VdW-df-cx,^31^ which has been previously employed to investigate charge transfer at the silicon–organic interface.^32^

The pseudopotential plane-wave calculations were performed using ultrasoft pseudopotentials.^33^ All the relaxation calculations were performed with a cutoff for the wave functions of 40 Ry and a 2 × 2 × 1 Monkhorst–Pack grid. We allowed a convergence of the total energy below 0.0001 Ry/atom. The systems were fully relaxed with a convergence threshold of 0.001 Ry/Å on the interatomic forces. The electronic structure calculations of the density of states (DOS) converged with 8 × 8 × 1 Monkhorst–Pack mesh of k-points.

We characterized the interfacial energetics of molecules ranging from C_2_ to C_10_ with single, double, and triple C–C bonds near the H–Si(111) surface. Specifically, we examined alkyl moieties (C_N_H_2N+1_) containing only single C–C bonds, 1-alkenyl moieties (C_N_H_N+1_) with the first C–C bond near the surface being a double bond followed by alternating single and double bonds, and 1-alkynyl moieties (C_N_H_2N-3_), where the first C–C bond near the surface is a triple bond, with the remaining C–C bonds being single.

The possible structural configurations of moiety adsorption onto the Si surface have been systematically explored by adopting a five-bilayer slab supercell model. The five-bilayer Si(111) slab is 15 Å thick; the box dimension perpendicular to the slab is set to *L*_*z*_ = 50 Å, creating a vacuum space to prevent interaction with adjacent images. The remaining dimensions are set to *L*_*x*_ = *L*_*y*_ = 15 Å. One moiety adsorbed per supercell corresponds to a density of 8.9 × 10^–19^ molecules/cm^2^.

**Figure 1:**
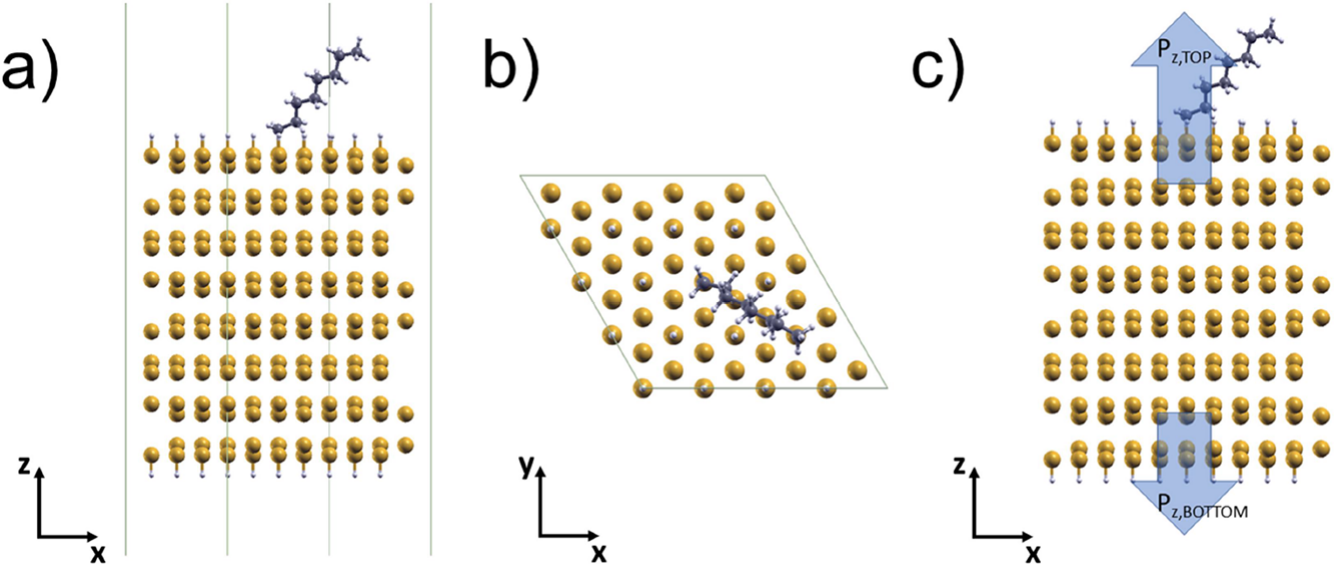
(a) Side and (b) top view of the eight-bilayer H–Si(111) slab model with adsorption of the C_N_ moiety on the top surface. (c) Dipoles associated with the bottom and top surfaces are shown. In this example, the C_8_ alkyl moiety is adsorbed. Yellow, white, and gray spheres are Si, H, and C atoms, respectively.

The most stable configurations on the five-bilayer slab (Figures S1–S3) have been used for an eight-bilayer slab supercell model (Figure 1a,b) to calculate the dipole variation with respect to the full hydrogenated surface upon relaxation of the extended structure. The eight-bilayer Si(111) slab is 23 Å thick; the box dimension perpendicular to the slab is set to *L*_*z*_ = 65 Å, and the remaining dimensions are set the same as for the five-bilayer slab. The chosen number of bilayers in both cases is a compromise between computational time and accuracy. The five-bilayer slab model is computationally cheaper and allows for exploration of many adsorption configurations. The slab models we used are asymmetric. Indeed, in the five-bilayer slab, the H atoms and the moiety were only adsorbed over the top layer, and the two bottom bilayers were fixed in the bulk positions (the bottom surface is clean); in the eight-bilayer slab, the top and the bottom layers were passivated by H atoms, the moiety was adsorbed over the top layer, and the two bilayers in the middle were fixed in the bulk positions. This required the correction for dipole self-interactions in the calculations.^34^

We calculated the total dipole along the *z* direction (perpendicular to the surface) of a system in the supercell as
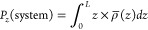
1where ρ̅(*z*) is the *xy*-plane integrated total charge density and *L* is the height of the supercell. We introduce the dipole Δ*P*_*z*_ that is the variation with respect to the fully H-passivated surface of the dipole along the *z* direction in consequence of the desorption of one H atom and the adsorption of the moiety. We calculate Δ*P*_*z*_ as the difference between the dipole *P*_*z*,TOP_ on the top surface (moiety chemisorbed on the H-passivated surface with substitution of one H atom) and the dipole *P*_*z*,BOTTOM_ of the bottom surface (fully H-passivated surface) projected along the *z* direction of the eight-bilayer slab. Therefore:
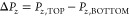
2where the *P*_*z*,TOP_ and *P*_*z*,BOTTOM_ dipoles are illustrated in Figure 1c. Using eq 1 within eq 2, it results that
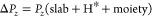
3where the slab + H* + moiety system is the supercell of the hydrogenated eight-bilayer slab with moiety adsorbed on top, as shown in Figure 1. The symbol H* means that one H has been removed from the adsorption site; e.g., slab + H* is the system (without the moiety), where all Si sites on the surface except the adsorption site are passivated with H. Therefore, eqs 1 and 3 allow us to calculate how much the dipole of the moiety adsorbed on the H-passivated surface changes with respect to the dipole of the fully H-passivated surface. On the other side, Δ*P*_mol_ is the difference between the dipole of the moiety bound to the surface in the full system, calculated as *P*_*z*_(slab + H* + moiety) – *P*_*z*_(slab + H*), and the dipole *P*_*z*_(moiety) of the isolated molecule, frozen in the geometry of adsorption. The dipole Δ*P*_mol_ quantifies the effect of the formation of the Si–C bond on the molecule; therefore, it is the dipole associated with the molecule–substrate bond. The positivity of Δ*P*_mol_ means an increase in the dipole of the molecule following the adsorption. Of course, the surface dipole is influenced by the tilting angle, which in turn depends on the specific adsorbate, as detailed in the following section on geometric structure.

## 3 Results and Discussion

### 3.1 Geometric Structure

**Figure 2:**
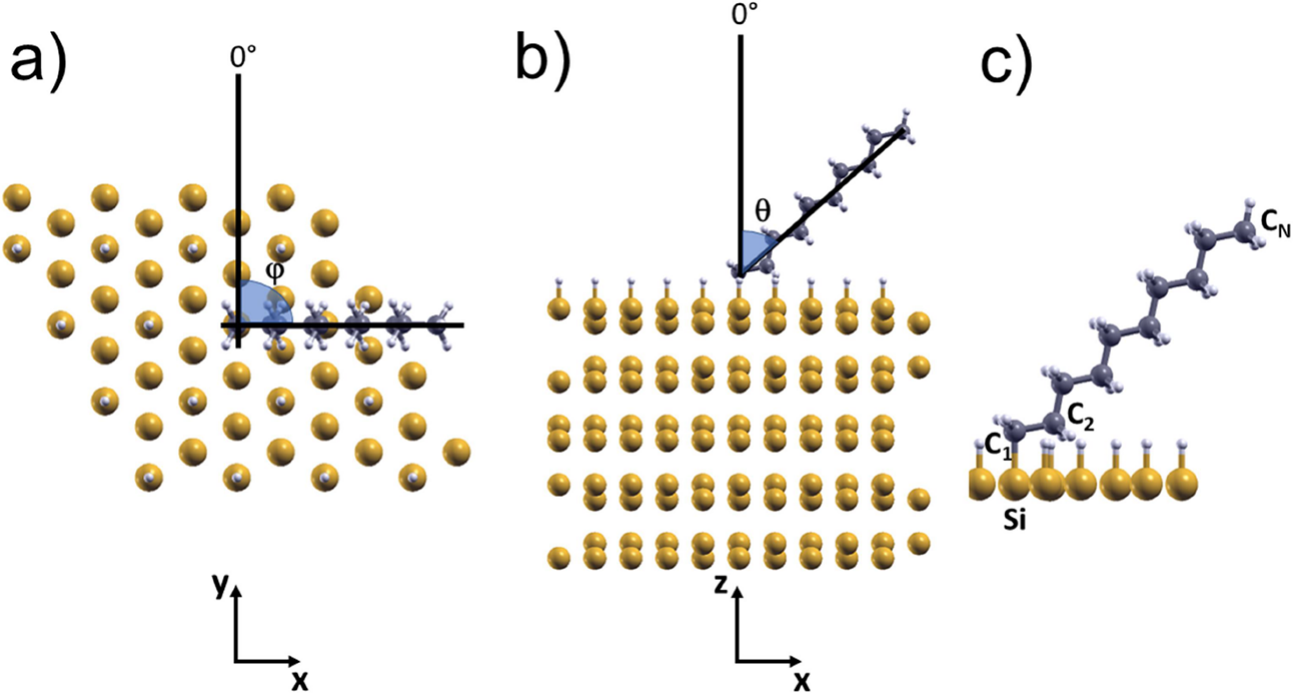
(a) Top and (b) side views of the H–Si(111) five-bilayer slab with an adsorbed aliphatic chain. The geometrical parameters φ and θ are indicated. (c) Detailed representation showing the **C**_**1**_, **C**_**2**_, **C**_**N**_, and **Si** atoms. Yellow, white, and gray spheres are Si, H, and C atoms, respectively.

We screened a wide range of possible adhesion configurations of molecules with single, double, and triple carbon–carbon bonds attached to the H–Si(111) surface and performed structural optimization for each of them. In the present work, we report the geometry corresponding to the minimal energy of adhesion, for which we have proceeded to the characterization of the electronic properties. We computed the molecule–surface dissociation energy as *E*_d_ = (*E*_m_ + *E*_s_) – *E*_sm_, where *E*_sm_ is the energy of the system with the molecule grafted onto the surface, and *E*_m_ and *E*_s_ are the energy of the isolated molecule and the surface, respectively. For each minimal energy configuration, structural characterization was conducted by calculating geometric parameters, including the bond distance *d*(**C**_**1**_**C**_**2**_) between the two carbon atoms closest to the surface and their respective bond angle θ(**C**_**1**_**C**_**2**_) with respect to the *z*-axis, the carbon–silicon bond distance *d*(**C**_**1**_**Si**), and the molecular axis bond angle with respect to the *z*-axis θ(**C**_**1**_**C**_**N**_), along with the molecule’s rotation angle φ in the xy plane (atomic labels and geometric parameters are referred in Figure 2). The values obtained are summarized in Table 1. In the following, the tilting angle θ(**C**_**1**_**C**_**N**_) is also simply referred to as the tilting angle. The description of the workflow process and the geometric structure parameters of the screened configurations can be found in the data set available on ref (35).

**Table 1:** Geometric Parameters of the Aliphatic Moieties Adsorbed on the H–Si(111) Surface^a^

moiety	*d*(**C_1_C_2_**)	*d*(**C_1_Si**)	θ(**C_1_C_N_**)	θ(**C_1_C_2_**)	φ	dissociation energy (eV)	group type
C_10_H_21_	1.526	1.904	36.32	64.02	14.77	3.827	alkyl
C_8_H_17_	1.529	1.905	36.41	62.09	119.01	3.829	alkyl
C_6_H_13_	1.527	1.906	48.16	68.02	134.12	3.791	alkyl
C_4_H_9_	1.528	1.907	42.34	62.51	141.83	3.792	alkyl
C_2_H_5_	1.529	1.910	57.79	57.79	109.92	3.761	alkyl
C_10_H_11_	1.360	1.860	34.76	56.35	72.02	4.505	1-alkenyl
C_8_H_9_	1.358	1.858	34.95	55.56	59.35	4.496	1-alkenyl
C_6_H_7_	1.357	1.864	41.15	59.47	169.68	4.449	1-alkenyl
C_4_H_5_	1.352	1.864	40.86	57.57	179.95	4.498	1-alkenyl
C_2_H_3_	1.339	1.873	50.54	50.54	174.95	4.204	1-alkenyl
C_10_H_17_	1.225	1.816	67.19	24.75	104.08	5.536	1-alkynyl
C_8_H_13_	1.224	1.813	58.78	22.82	46.57	5.462	1-alkynyl
C_6_H_9_	1.223	1.811	37.74	16.53	119.10	5.442	1-alkynyl
C_4_H_5_	1.223	1.812	35.48	9.25	148.46	5.415	1-alkynyl
C_2_H	1.219	1.824	1.98	1.98	150.82	5.506	1-alkynyl

a Refer to Figure 2 for the meaning of the listed parameters and atomic labels.

The *d*(**C**_**1**_**Si**) and *d*(**C**_**1**_**C**_**2**_) bond lengths are decreasing in the following order: alkyl, 1-alkenyl, 1-alkynyl. To describe how the bond lengths change with respect to the group type, we can approximately state that *d*(**C**_**1**_**Si**) ≈ 1.9, 1.85, and 1.8 Å and *d*(**C**_**1**_**C**_**2**_) ≈ 1.5, 1.35, and 1.2 Å for alkyl, 1-alkenyl, and 1-alkynyl moieties, respectively.

The tilting angle of the alkyl and 1-alkenyl moieties' **C**_**1**_**C**_**2**_ bond between the two C atoms closest to the surface is always higher (i.e., more tilted) than that of the molecular axis **C**_**1**_**C**_**N**_. For example, in the case of C_10_ chains, the alkyl moiety has θ(**C**_**1**_**C**_**2**_) = 64.02° and θ(**C**_**1**_**C**_**N**_) = 36.32°, and the 1-alkenyl moiety has θ(**C**_**1**_**C**_**2**_) = 56.35° and θ(**C**_**1**_**C**_**N**_) = 34.76°. The opposite happens for the 1-alkynyl moiety that has θ(**C**_**1**_**C**_**2**_) = 24.75° and θ(**C**_**1**_**C**_**N**_) = 67.19° for the C_10_ example.

The tilting of the molecular axis **C**_**1**_**C**_**N**_ of alkyl moieties smoothly changes with respect to the carbon chain length for *N* > 2, and then, it is almost constant for *N* = 8 and 10 with θ(**C**_**1**_**C**_**N**_) ≈ 36°. The same holds for 1-alkenyl moieties, where θ(**C**_**1**_**C**_**N**_) reaches the value of 35° for *N* = 8 and 10. Instead, the tilting of 1-alkynyl moieties increases with respect to *N* with 2° < θ(**C**_**1**_**C**_**N**_) < 68°. In particular, the 1-alkynyl moieties show the following peculiar behavior: the shortest carbon chains are not very tilted in the sense that they are almost perpendicular to the surface, for example, θ(**C**_**1**_**C**_**N**_) = 2°, 35°, and 38° (the lowest tilting angles) for C_2_, C_4_, and C_6_, respectively; the longest carbon chains of 1-alkynyl moieties have the highest tilting angle across the three chemical groups, for example, θ(**C**_**1**_**C**_**N**_) = 59° and 67° for C_8_ and C_10_ chains, respectively. The difference in tilting compared to other aliphatic groups can be attributed to the rigidity of the triple bond, which consists of one σ bond and two π bonds. This structural rigidity results in lower θ(**C**_**1**_**C**_**2**_) angles for alkynyl moieties, as shown in Table 1. The trends of the tiling angle increasing with respect to *N* agree with the findings of Yuan et al.^22^ We found a weak dependence of the total energy on the molecule’s rotation angle φ in the *xy* plane.

The 1-alkynyl moieties have a higher dissociation energy, above 5.5 eV, followed by the 1-alkenyl moieties with dissociation energies between 4.2 (C_2_H_3_) and 4.5 (C_10_H_11_) eV. The alkyl moieties have a lower dissociation energy, around 3.8 eV.

### 3.2 Dipole Formation and Schottky Barriers

A surface dipole is generated when the aliphatic chain is adsorbed on the silicon surface; the Si–C bond is formed, accompanied by charge transfer between the adsorbate and the surface. Considering the Pauling electronegativity of Si (1.90) and C (2.5), a net electron density would be expected around the carbon atom of Si–C, resulting in a negative surface dipole (i.e., negative charge pointing outward and positive charge pointing inward). Instead, based on our calculations, we will show that the resulting surface dipole is positive, and not negative, as predicted from electronegativity considerations on the Si–C bond only. In the following, we demonstrate that the effect of the Si–C bond formation on the whole chain must be considered to find the correct dipole.

The dipole Δ*P*_*z*_ formed upon the desorption of one H atom and the adsorption of the moiety has been calculated based on eqs 1 and 3. The energy steps ψ and ψ_mol_ associated with the dipoles Δ*P*_*z*_ and Δ*P*_mol_, respectively, are expressed by the following equations:^36^
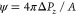
4
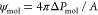
5where *A* is the surface area of the supercell. The energy step ψ is the change of the work function induced by the dipole. In eqs 4 and 5, we see that the dipole expressed in eV is intimately related to the adsorbate density on the surface. Therefore, for a fixed value of the dipole, decreasing the surface per adsorbate, we obtain a higher energy step.

In the following, the surface dipole is quantified as the energy step ψ corresponding to one adsorbate per supercell. We found that the surface dipole of alkyl moieties, reported in Table 2, changes from 0.115 to 0.153 eV, varying within a range as short as 0.038 eV, and from 0.103 to 0.324 eV for the 1-alkenyl moieties (Table 3), changing in a range of 0.221 eV. Therefore, increasing the length of the 1-alkenyl moieties from C_2_ to C_10_ results in an overall variation of the dipole five times larger than that of the alkyl moieties.

**Table 2:** Ab Initio Dipoles Δ*P*_*z*_ and Δ*P*_mol_, the ψ and ψ_mol_ Energy Steps, and Charge Transfer of Alkyl Moieties Adsorbed on the H–Si(111) Surface^a^

chain	Δ*P*_*z*_ (D)	ψ (eV)	Δ*P*_mol_ (D)	ψ_mol_ (eV)	charge transfer (e)
C_2_	0.633	0.115	0.707	0.129	0.12
C_4_	0.775	0.141	0.582	0.106	0.12
C_6_	0.826	0.150	0.577	0.105	0.12
C_8_	0.823	0.150	0.521	0.095	0.11
C_10_	0.839	0.153	0.454	0.083	0.11

a The energy steps refer to one moiety adsorbed per supercell (see text).

**Table 3:** Ab Initio Dipoles Δ*P*_*z*_ and Δ*P*_mol_, the ψ and ψ_mol_ Energy Steps, and Charge Transfer of 1-Alkenyl Moieties Adsorbed on the H–Si(111) Surface^a^

chain	Δ*P*_*z*_ (D)	ψ (eV)	Δ*P*_mol_ (D)	ψ_mol_ (eV)	charge transfer (e)
C_2_	0.567	0.103	0.320	0.058	0.18
C_4_	0.947	0.172	0.647	0.118	0.17
C_6_	1.248	0.227	0.931	0.169	0.16
C_8_	1.505	0.274	0.545	0.099	0.16
C_10_	1.784	0.324	0.115	0.021	0.15

a The energy steps refer to one moiety adsorbed per supercell (see text).

The dipole Δ*P*_mol_ of the alkyl moieties has a small dependence on the length of molecules; indeed, the energy step ψ_mol_ (calculated by the eq 5) assumes values from 0.083 to 0.129 eV. This means a small increase in the molecular dipole upon adsorption. On the other side, ψ_mol_ of the 1-alkenyl moieties varies in a range from 0.021 to 0.169 eV, wider than the one for alkyl moieties. This means that the double C–C bond induces a more pronounced molecular dipole response upon adsorption. The tilting angle varies within a narrow range of 15° for alkyl and 10° for 1-alkenyl moieties, suggesting that its influence on dipole is minimal.

In contrast to alkyl and 1-alkenyl, the 1-alkynyl moieties exhibit a nonmonotonic variation of the dipole (Table 4). Indeed, starting from the value of 0.112 eV for C_2_H, ψ increases to 0.296 and 0.295 eV for C_4_H_5_ and C_6_H_9_ and then decreases to 0.264 and 0.257 eV for C_8_H_13_ and C_10_H_17_, respectively. Therefore, ψ increases by 0.184 eV moving from C_2_H to C_4_H_5_ 1-alkynyls, and then, it remains almost constant with respect to the length of the molecule, showing a variation of just 0.039 eV. However, the overall dipole variation of 0.184 eV for 1-alkynyls is comparable to that of 1-alkenyl moieties. In this case, the tilting angle θ(**C**_**1**_**C**_**N**_) varies in a wide range from 2° to 67° by increasing *N*, strongly influencing the dipole on the surface. It is useful to see how adsorption affects the dipole of the isolated moiety, also for 1-alkynyl moieties. When we consider the dipole associated with the molecule–substrate bond, the dipole Δ*P*_mol_ of 1-alkynyl adsorbates changes with respect to *N* in the opposite direction of alkyl and 1-alkenyl moieties (Table 4). Indeed, ψ_mol_ is −0.118 and −0.160 eV for C_2_H and C_10_H_17_ 1-alkynyl moieties, respectively, reaching the largest value of −0.214 eV for the C_6_H_9_ 1-alkynyl moiety. Therefore, the dipole of the 1-alkynyl moiety adsorbed on H–Si(111) decreases when compared to the same moiety considered as isolated. To understand this different behavior with respect to the other moiety group types, the charge analysis is required.

**Table 4:** Ab Initio Dipoles Δ*P*_*z*_ and Δ*P*_mol_, the ψ and ψ_mol_ Energy Steps, and Charge Transfer of 1-Alkynyl Moieties Adsorbed on the H–Si(111) Surface^a^

chain	Δ*P*_*z*_ (D)	ψ (eV)	Δ*P*_mol_ (D)	ψ_mol_ (eV)	charge transfer (e)
C_2_	0.616	0.112	–0.650	–0.118	0.29
C_4_	1.628	0.296	–1.044	–0.190	0.25
C_6_	1.620	0.295	–1.179	–0.214	0.24
C_8_	1.449	0.264	–1.021	–0.186	0.22
C_10_	1.415	0.257	–0.881	–0.160	0.21

a The energy steps refer to one moiety adsorbed per supercell (see text).

**Figure 3:**
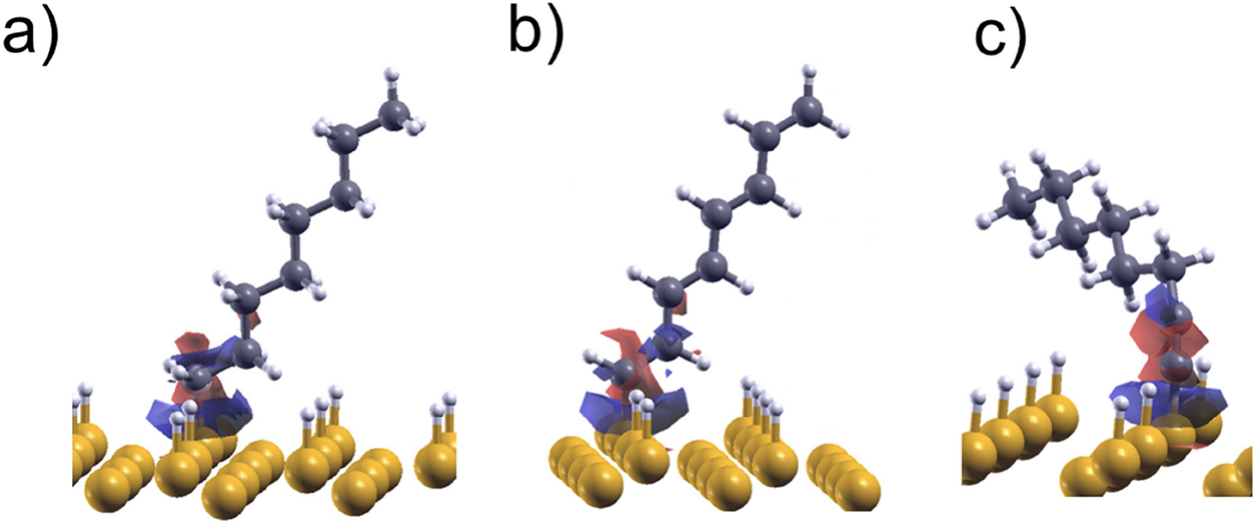
Difference in charge density of (a) alkyl, (b) 1-alkenyl, and (c) 1-alkynyl C_8_ moieties adsorbed on the H–Si(111) surface (yellow, white, and gray spheres are Si, H, and C atoms, respectively; red and blue are negative and positive isosurfaces, respectively; the isosurface value is ± 0.003 au).

To better understand the interaction between the substrate and the aliphatic moiety, we have calculated Löwdin charges and the difference in charge density. The negative charge transferred from the substrate to the moiety is in the range 0.11–0.12 *e* for alkyl, 0.15–0.18 *e* for 1-alkenyl, and 0.21–0.29 *e* for 1-alkynyl moieties. As a rule, we found that the charge transfer increases with the order of the C–C bond close to the surface. Indeed, the localization of the electrons increases with the bond order, as shown by the difference in charge density in Figure 3c, where C_8_ adsorbates are considered; we see that more negative charge is accumulated around the triple bond close to the surface of 1-alkynyls (the same considerations hold for 1-alkynyl chains with different *N*). This negative charge partially compensates the positive charge of the tail, and it causes a decrease in the dipole of 1-alkynyl moieties with respect to the isolated ones so that Δ*P*_mol_ is negative.

**Figure 4:**
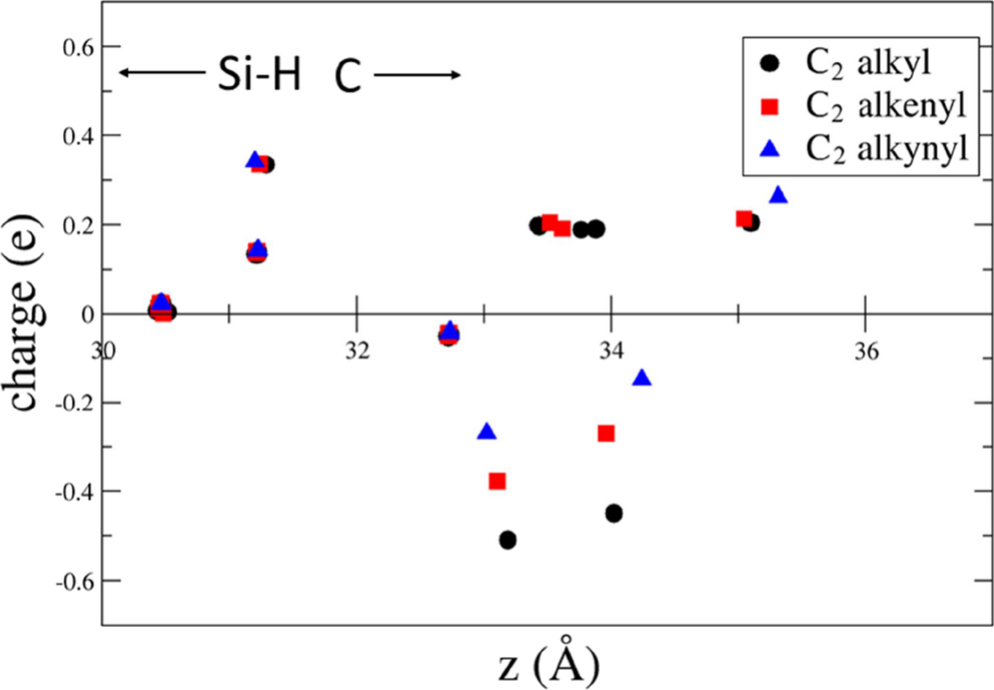
Difference between the Löwdin charge and the neutral atomic charge as a function of the *z* coordinate perpendicular to the surface for the C_2_ alkyl, 1-alkenyl, and 1-alkynyl moieties.

**Figure 5:**
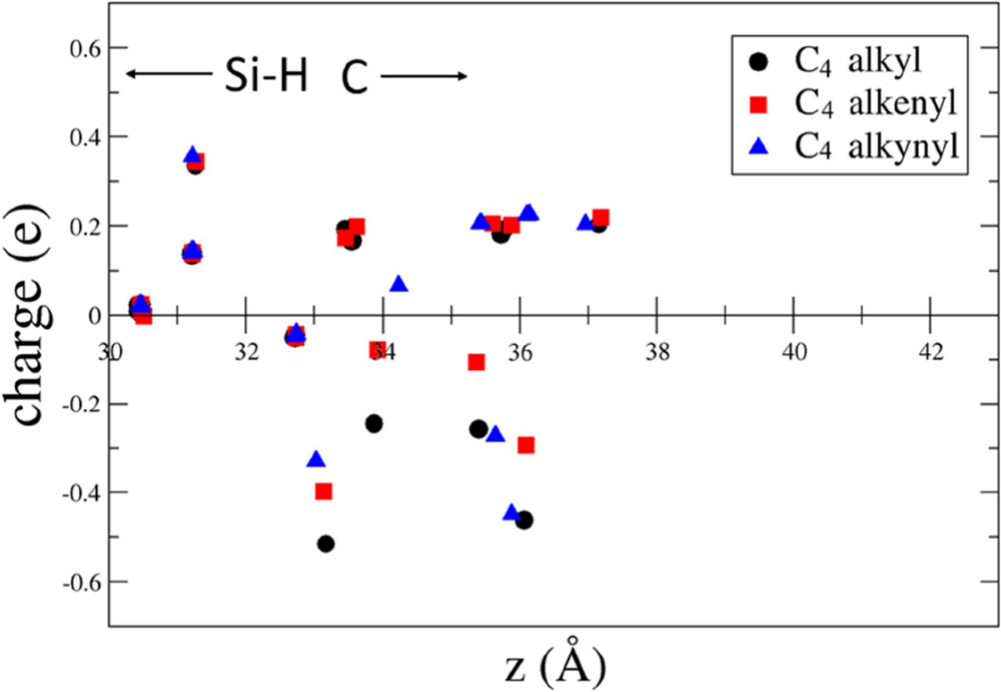
Difference between the Löwdin charge and the neutral atomic charge versus the *z* coordinate perpendicular to the surface for the C_4_ alkyl, 1-alkenyl, and 1-alkynyl moieties.

**Figure 6:**
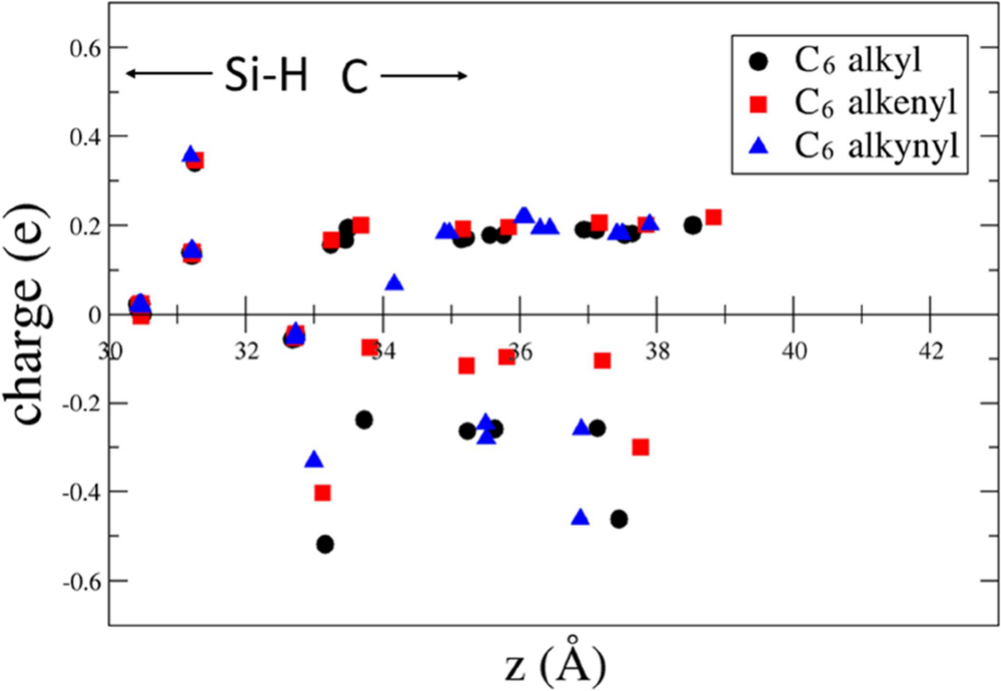
Difference between the Löwdin charge and the neutral atomic charge versus the *z* coordinate perpendicular to the surface for the C_6_ alkyl, 1-alkenyl, and 1-alkynyl moieties.

**Figure 7:**
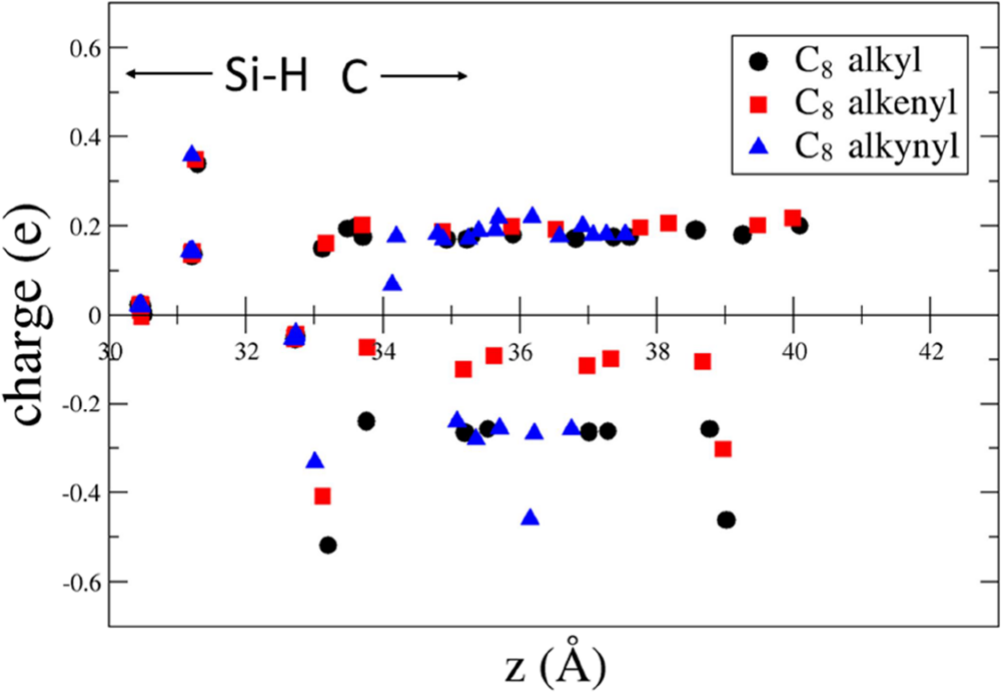
Difference between the Löwdin charge and the neutral atomic charge versus *z* coordinate perpendicular to the surface for the C_8_ alkyl, 1-alkenyl, and 1-alkynyl moieties.

**Figure 8:**
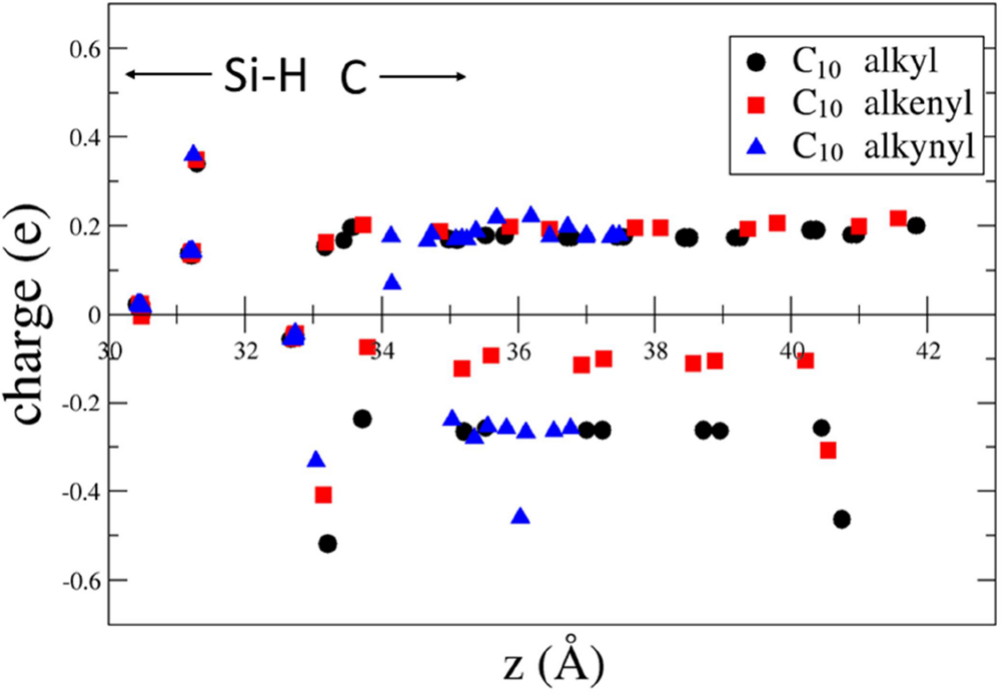
Difference between the Löwdin charge and the neutral atomic charge versus the *z* coordinate perpendicular to the surface for the C_10_ alkyl, 1-alkenyl, and 1-alkynyl moieties.

To gain more insights into the charge distribution along the chain, we have plotted in Figures 4–8 the difference between the Löwdin and the neutral atomic charges as a function of the z coordinate, perpendicular to the surface, for chains ranging from C_2_ to C_10_ of the three moieties groups. Given the presence of the bottom vacuum slab, the surface is located at approximately *z* = 30 Å. It is evident that a positive charge is localized on the top bilayer Si atom, and a negative charge is located on the atoms of the molecule close to the surface. The charge oscillates along the chain, moving upward along the moiety, and then, a positive charge is localized on the terminal group opposite to the surface. This distribution of charges results in the positive dipole (dipole pointing outward). The presence of negative partial charges on carbon atoms and positive partial charges on hydrogen atoms is consistent with the higher electronegativity of carbon relative to hydrogen. As we stated above, basic considerations based on Pauling electronegativity suggest that a net electron density accumulates near the carbon atoms in the Si–C bond, resulting in the formation of a negative surface dipole. Indeed, the calculation of the Löwdin charges shows that negative charges move from the surface to the C of the Si–C bond, indicating electron gain, leaving positive charges around the Si atom (i.e., electron loss). However, the resulting surface dipole is not only related to the Si–C bond. When the dipole induced by the adsorption of the aliphatic chain is calculated, it is found to be positive, contrary to the expectation based on having considered only the C atom closer to the surface. This discrepancy can be readily explained by recognizing that, following the removal of a hydrogen atom from one of the molecule’s terminal groups and the subsequent formation of the Si–C bond, the molecule becomes asymmetric, and the charges located on the opposite terminal group (−CH_3_ for alkyl and 1-alkynyl and −CH_2_ for 1-alkenyl moieties) contribute to the overall dipole. Our calculations demonstrate that the charge localized on the moiety’s terminal group furthest from the surface is positive, thereby leading to the positive dipole induced by the adsorption of the moiety.

In Figures 4–8, it is also evident that the distance of the terminal group opposite to the surface from the interface of 1-alkynyl moieties decreases with respect to the number of C atoms. This is connected to the enhancement of the tilting angle as an effect of the triple C–C bond close to the surface, as we have discussed above.

**Figure 9:**
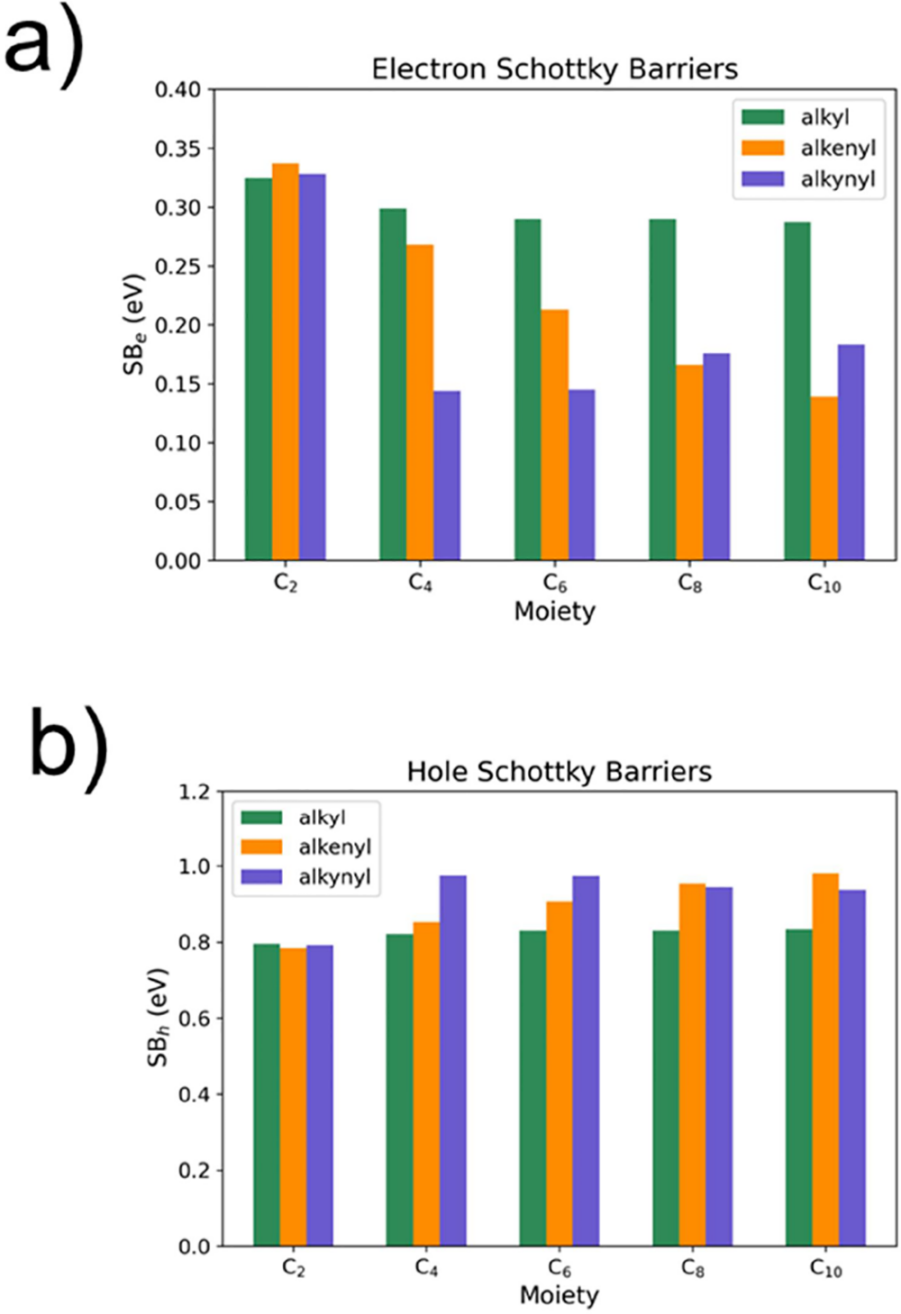
(a) Electron and (b) hole Schottky barriers of the alkyl, 1-alkenyl, and 1-alkynyl modified Hg/H–Si(111) heterojunction calculated for the one moiety adsorbed per supercell model.

The dipole formed at the interface affects the thermionic transport at the silicon/organic molecule/metal heterojunction because of the Schottky barrier (SB). Indeed, the SB depends on the surface dipole as follows^10,16^:
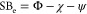
6
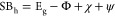
7where Φ is the work function of the metal, χ is the electronic affinity of the semiconductor (Si), ψ is the energy step of the surface dipole in eq 4, and E_g_ is the band gap of the semiconductor. SB_e_ and SB_h_ are the SBs at zero bias of electrons and holes, respectively. From eqs 6 and 7, we see that positive surface dipoles reduce the SB of electrons and enhance the SB of holes. Upon eqs 6 and 7 and the values of the surface dipole reported in Tables 2–4, we can estimate how the SB depends on type and length of the aliphatic moiety. In Figure 9, we show how the electron and hole SBs of the Si–H/molecule/metal heterojunction change by varying the adsorbed moiety when the metal is Hg by assuming Φ_Hg_ = 4.5 eV, χ_Si_ = 4.06 eV,^37^ and E_g_ = 1.12 eV. When the adsorbate is an alkyl moiety, a slow variation of the SB is found with respect to N, with SB_e_ ≈ 0.3 eV and SB_h_ ≈ 0.8 eV. Attaching 1-alkenyl moieties, a monotonic and significant variation of the SB is seen. For the adsorbate density of the interface in Figure 1, SB_e_ (SB_h_) varies from 0.34 to 0.14 (0.78 to 0.98) eV for the chains with *N* = 2 and *N* = 10, respectively. We have different findings for 1-alkynyls. Specifically, SB_e_ (SB_h_) of the C_2_H 1-alkynyl moiety is 0.33 (0.79) eV. A change of 0.2 eV is obtained for C_4_ and C_6_, followed by a slow variation of 0.04 eV for C_8_ and C_10_ (decrease of SB_e_ and increase of SB_h_). To resume, among the investigated range of aliphatic chains, only 1-alkenyl moieties exhibit a regular and significant variation of the SB by increasing *N* of the moiety. This is important when the electronic energetics at the interface must be tuned to control the thermionic transport in electronic devices. Of course, following eq 4, by increasing the density of adsorbates, the impact of the molecular dipole on the SB is enhanced. The qualitative variation of the SB for alkyl and 1-alkenyl moieties (i.e., SB_e_ decreases by increasing the length of the molecular chain) agrees with the experimental findings of Har-Lavan et al.^38^

### 3.3 Interface Energetics and Tunneling Barriers

**Figure 10:**
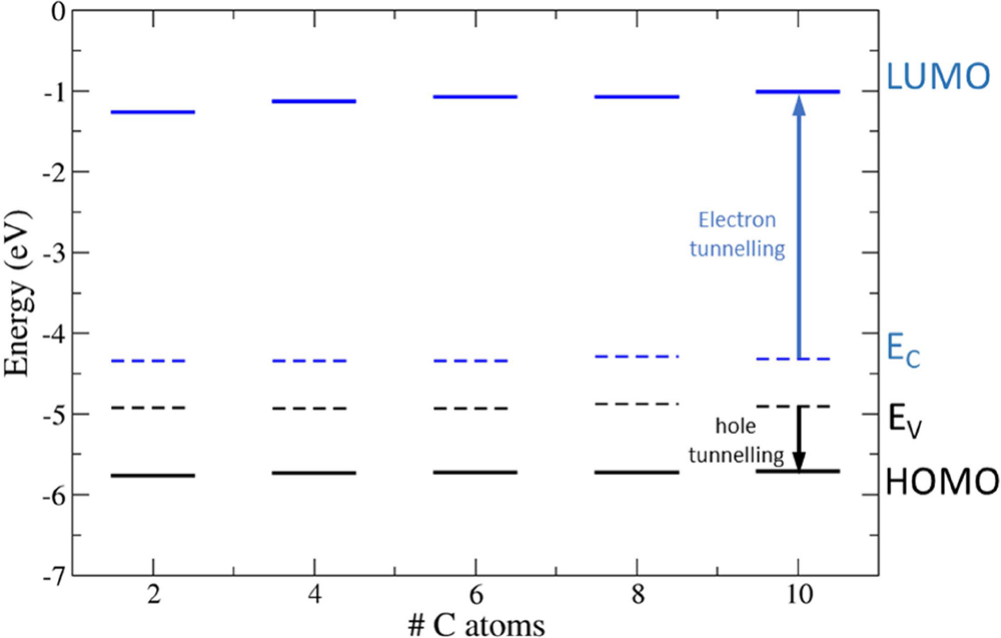
Valence (*E*_V_) and conduction (*E*_C_) band edges (dashed lines) of bulk silicon and HOMO/LUMO energy levels (full lines) of alkyl moieties (black: occupied states; blue: unoccupied states). The electron and hole tunneling barriers are evidenced as blue and black arrows, respectively, for the C_10_ chain. The vacuum level is set to 0 eV.

**Figure 11:**
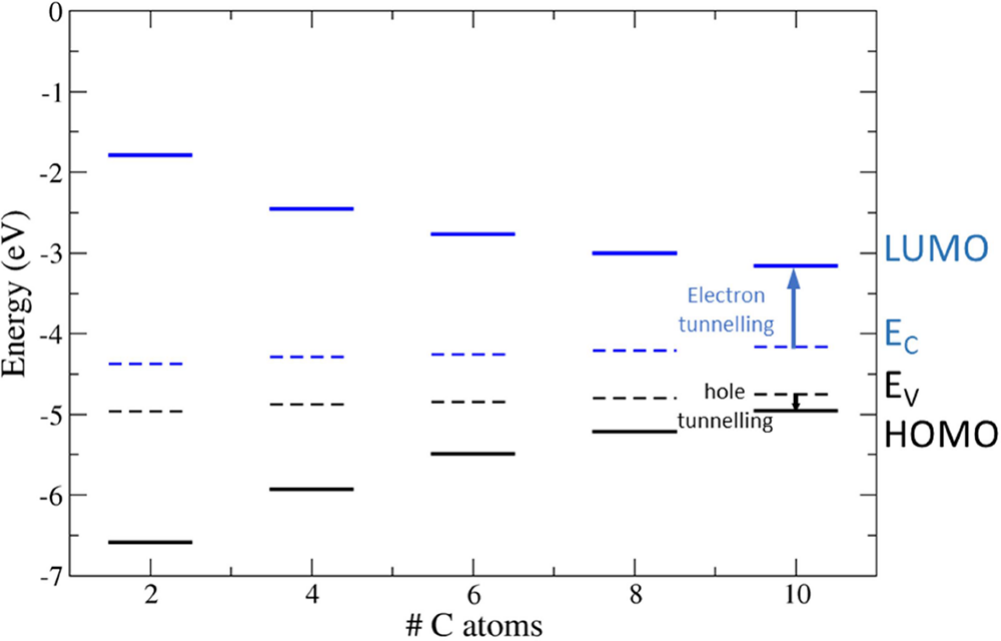
Valence (*E*_V_) and conduction (*E*_C_) band edges (dashed lines) of bulk silicon and HOMO/LUMO energy levels (full lines) of 1-alkenyl moieties (black: occupied states; blue: unoccupied states). The electron and hole tunneling barriers are evidenced as blue and black arrows, respectively, for the C_10_ chain. The vacuum level is set to 0 eV.

**Figure 12:**
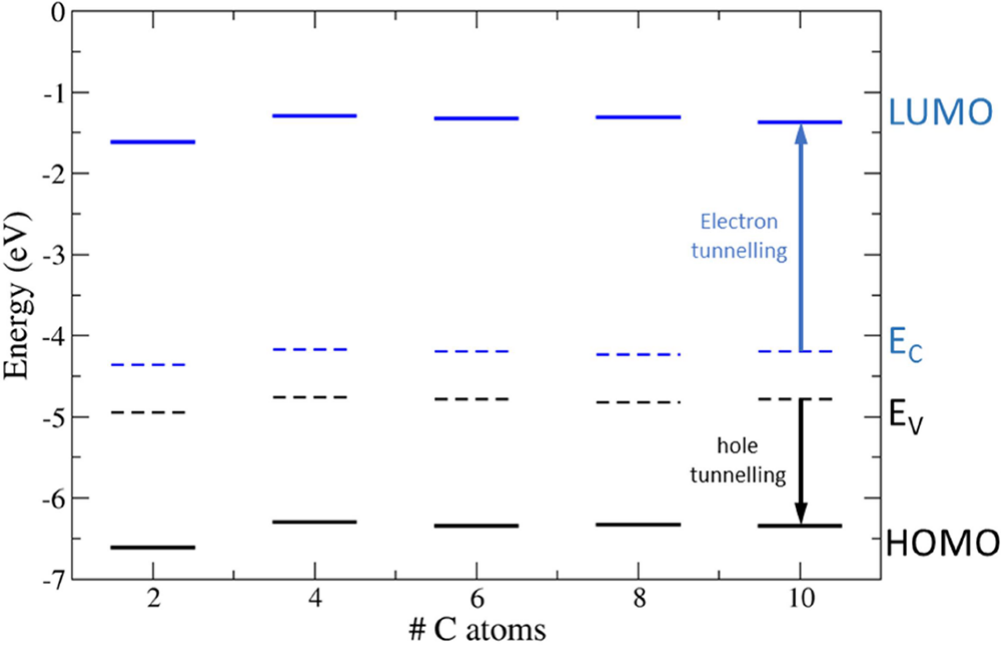
Valence (*E*_V_) and conduction (*E*_C_) band edges (dashed lines) of bulk silicon and HOMO/LUMO energy levels (full lines) of 1-alkynyl moieties (black: occupied states; blue: unoccupied states). The electron and hole tunneling barriers are evidenced as blue and black arrows, respectively, for the C_10_ chain. The vacuum level is set to 0 eV.

We determined the band edges and the frontier orbital levels to investigate the energetics of the H–Si(111)/aliphatic chain heterointerface. We estimated the vacuum level of each adsorption configuration as the constant plane-averaged electrostatic energy in the vacuum gap far away from the top atomic layer, as shown in Figure S4. The valence and conduction band edges of the bulk silicon have been aligned to those of the silicon slab by overlapping the plane-averaged electrostatic energy in the central layers. We determined the HOMO and LUMO energies of the aliphatic moieties as the peaks of the projected DOS (PDOS) of the atomic orbitals associated with the organic molecule near the band gap (PDOS are shown in Figures S5–S19). The frontier orbital energy levels of alkyl, 1-alkenyl, and 1-alkynyl moieties are compared to the band edges of bulk Si. The evolution of the energy levels is shown in Figures 10–12 as the C chain extends from C_2_ to C_10_. The same data are reported in Tables S1–S3. The energy shifts of HOMO and LUMO with respect to valence and conduction band edges (*E*_V_ and *E*_C_, respectively) are shown in Tables 5–7 and indicated as ΔHOMO (=*E*_V_ – HOMO) and ΔLUMO (=LUMO – *E*_C_), respectively. ΔHOMO and ΔLUMO are the energy barriers for the tunneling of holes and electrons, respectively. Of course, the tunneling current is also influenced by the length of the molecular bridge.

**Table 5:** Ab Initio Tunneling Barriers for Holes (ΔHOMO) and Electrons (ΔLUMO) of the Alkyl Moieties Adsorbed on the H–Si(111) Surface

chain	ΔHOMO (eV)	ΔLUMO (eV)
C_2_	0.83	3.08
C_4_	0.80	3.21
C_6_	0.79	3.28
C_8_	0.83	3.23
C_10_	0.81	3.31

**Table 6:** Ab Initio Tunneling Barriers for Holes (ΔHOMO) and Electrons (ΔLUMO) of the 1-Alkenyl Moieties Adsorbed on the H–Si(111) Surface

chain	ΔHOMO (eV)	ΔLUMO (eV)
C_2_	1.63	2.59
C_4_	1.05	1.84
C_6_	0.65	1.49
C_8_	0.41	1.21
C_10_	0.21	1.01

**Table 7:** Ab Initio Tunneling Barriers for Holes (ΔHOMO) and Electrons (ΔLUMO) of the 1-Alkynyl Moieties Adsorbed on the H–Si(111) Surface

chain	ΔHOMO (eV)	ΔLUMO (eV)
C_2_	1.67	2.75
C_4_	1.54	2.88
C_6_	1.56	2.87
C_8_	1.51	2.93
C_10_	1.56	2.83

The energy gap of the adsorbed alkyl and 1-alkynyl moieties has a weak dependence on the length of the chain. Indeed, the HOMO energy level of alkyls is almost independent of the moiety, while the LUMO energy level is a little bit more affected; ΔLUMO varies in a range of 0.23 eV centered around 3.2 eV that is wider than the variation of 0.04 eV of ΔHOMO centered around 0.81 eV. Conversely, the energy variation changing the number of C atoms for occupied and unoccupied orbitals of the 1-alkynyl moieties is similar because ΔLUMO and ΔHOMO vary in a range of 0.18 and 0.16 eV, centered around 2.8 and 1.6 eV, respectively. Comparing alkyls and 1-alkynyls, we found that alkyl (1-alkynyl) moieties have the lowest hole (electron) energy barrier for tunneling.

On the other side, the energy shifts of HOMO and LUMO of 1-alkenyl moieties are more sensitive to the length of the chain. The reason for this is that the energy gap of 1-alkenyls shrinks from 4.8 (C_2_H_3_) to 1.8 (C_10_H_11_) eV by increasing *N*, so that the frontier orbital energy levels get closer and closer to the band edges nearby. The decrease in the energy gap of alkenyl moieties as *N* increases is attributed to the enhanced electron delocalization with the growing number of double bonds along the chain. This involves the values of ΔLUMO (ΔHOMO) of 1-alkenyl adsorbates changing from 1.0 to 2.6 (0.2–1.6) eV, the variation being in a wide range of 1.41 (1.58) eV. The 1-alkenyl moieties originate in absolute to the lowest energy barriers by increasing the length of the molecular chain. The adsorption of the C_10_ 1-alkenyl moiety reduces the tunneling barrier the most and enhances transport related to this charge transfer mechanism. Although nonlocal functionals still underestimate the energy gap of molecules and semiconductors, it is interesting to observe that the electron tunneling barrier of C_9_ 1-alkenyl moiety in the experiments is found to be around 1 eV,^39^ in nice agreement with the values of 1.2 and 1.0 eV for C_8_ and C_10_ 1-alkenyl moieties, respectively, obtained in the present work.

## 4 Conclusions

In the present work, we have investigated the effects of adsorption of single aliphatic moieties on H–Si(111) surfaces. A wide range of possible adhesion configurations of alkyl (C_*n*_H_2*n*+1_), 1-alkenyl (C_*n*_H_*n*+1_), and 1-alkynyl (C_*n*_H_2*n*–3_) moieties has been explored, and the structural characterization in the minimal energy geometries has been reported. The presence of single, double, or triple C–C bonds near the surface has different impacts on the silicon/molecule interface energetics. The 1-alkynyl moieties have a higher dissociation energy, followed by the 1-alkenyl and alkyl moieties. The trend of the tilting of the molecular axis **C**_**1**_**C**_**N**_ of 1-alkynyl moieties is to increase with the number of C atoms. Indeed, the shortest 1-alkynyl adsorbate is almost perpendicular to the surface, while the longest one exhibits the highest tilting angle.

The surface dipole influences the SB at the semiconductor/organic/metal interface, given the work function of the metal and the electronic affinity of the semiconductor, and affects the thermionic transport. Simple considerations on the Pauling electronegativity of the Si–C bond wrongly predict the surface dipole to be negative. Rather, to correctly predict the direction of the dipole, the distribution of the charge along the entire aliphatic chain should be considered. We showed that the positive charge localized on the moiety’s terminal group opposite to the surface gives rise to the positive dipole (i.e., positive charge pointing outward and negative charge pointing inward). The alkyl and 1-alkynyl adsorbates give almost constant SB when the aliphatic chain is longer than *N* = 2. Instead, the 1-alkenyl moieties exhibit a regular and significant variation of the SB by increasing *N* of the hydrocarbon chain. In summary, short aliphatic chains lead to high electron SBs and low hole SBs, which play a crucial role in tuning the electronic energetics at the interface. The optimal molecular choice depends on whether an insulating or conductive interface is desired.

We calculated the alignment of the frontier molecular orbital energy levels to the bands of the semiconductor to get insights into the tunneling transport. Comparing alkyl and 1-alkynyl moieties, we found that the alkyl (1-alkynyl) chains have the lowest hole (electron) energy barrier for tunneling, with weak dependence on *N*. Instead, the 1-alkenyl moieties give rise in absolute to the lowest energy barriers, whose height decreases by increasing *N*. Indeed, the C_10_ 1-alkenyl moiety reduces the tunneling energy barrier to 1 eV for electrons and 0.2 eV for holes, and we predict it to enhance the most the tunneling mechanism. The range of values we found is in good agreement with the available experimental measurements. In monolayers, broadening of the energy levels is present due to the interaction between adsorbates and the substrate, as well as among molecules.

The applicability of our approach and findings extends beyond thermionic and tunneling transport in electronic devices. Indeed, our results can also be useful in other contexts, such as tuning of electron or hole transport layers in photovoltaic devices or regulation of the interface energetics in water-splitting applications. The present study can be expanded by pursuing the following objectives: investigation of longer aliphatic chains until C_18_; adsorption on other relevant Si surfaces as Si(110) and Si(100); and usage of the ab initio calculated parameters as the input of phenomenological models. The findings for single moiety adsorption presented in this work pave the way to further studies targeting the adsorption of multiple moieties forming monolayers. This target holds significant implications for the development of next-generation electronics, sensors, and photovoltaic systems, highlighting the potential of aliphatic chain surface functionalization in enhancing silicon’s already impressive capabilities.

## Supporting Information Available

Minimal energy configurations; plane-averaged electrostatic energy; projected density of states (PDOS); and ab initio calculated energy levels (PDF)
