# Supplementary material for: Computational Insights into the Energetics of Single C2–C10 Aliphatic Moieties Adsorbed on the Hydrogenated Silicon (111) Surface
Source: Langmuir. 2025 Apr 8;41(15):9706–15. doi: 10.1021/acs.langmuir.4c05103 (PMC12020416; doi:10.1021/acs.langmuir.4c05103)
Supplement: Supplementary file 1 — la4c05103_si_001.pdf [file la4c05103_si_001.pdf]

Computational Insights into the Energetics of Single C_2-C_{10} Aliphatic Moieties Adsorbed on Hydrogenated Silicon (111) Surface

Francesco Buonocore [a*], Sara Marchio [a], Simone Giusepponi [a], Massimo Celino [a]

[a] Italian National Agency for New Technologies, Energy and Sustainable Economic Development (ENEA) − C. R. Casaccia, Via Anguillarese 301, 00123 Rome, Italy
* Email: francesco.buonocore@enea.it

Abstract. Silicon's versatility as a semiconductor renders it indispensable across various domains, including electronics, sensors, and photovoltaics. Modifying hydrogen-terminated silicon surfaces with moieties adsorption offers a method to tailor the material's properties for specific applications. In this study, we employ ab initio density functional theory calculations to explore the energetics of single alkyl, 1-alkenyl and 1-alkynyl moieties chemisorbed on hydrogen-terminated silicon (111) surface. We analyse the interfacial dipole induced by Si–C bond formation that determines the Schottky barrier and examine the alignment of the frontier orbitals energy levels with silicon band structure to investigate the charge transfer based on tunnelling mechanism. Our findings provide valuable insights into how aliphatic moiety functionalization affects interfacial electronic properties, offering clues for optimizing silicon-based devices.

Computational Insights into the Energetics of Single C_2-C_{10} Aliphatic Moieties Adsorbed on Hydrogenated Silicon (111) Surface

I. Supporting Information

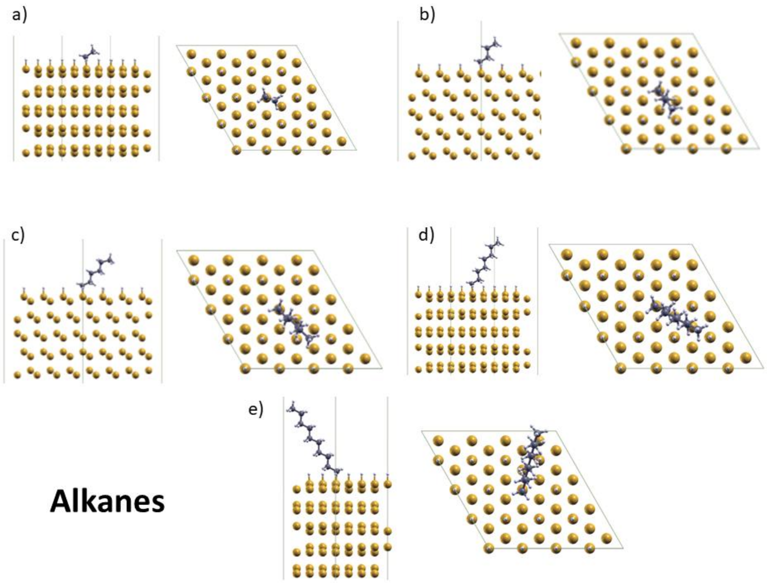

Figure S1. Side and top view of relaxed coordinates of a) C_2; b) C_4; c) C_6; d) C_8 and e) C_{10} alkane chains adsorbed on the five bilayers H−Si(111) slab (H atoms and the moiety only adsorbed over the top layer) in the most stable adsorption configurations.

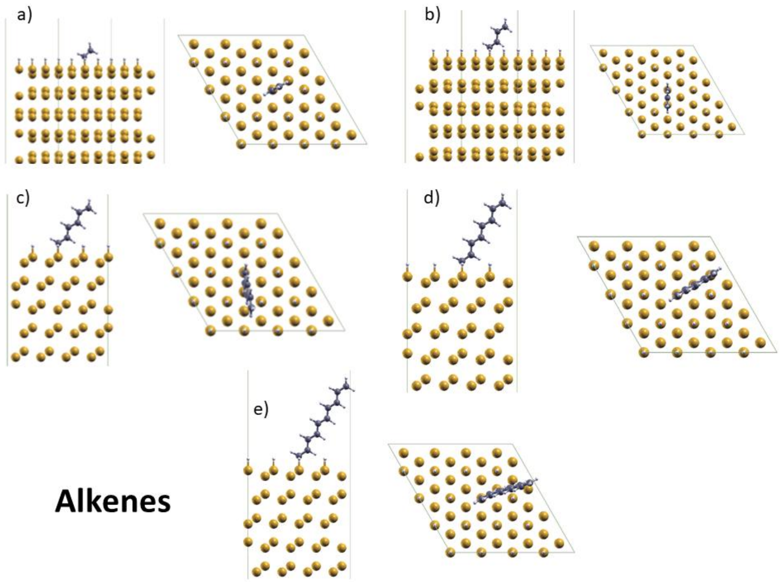

Figure S2. Side and top view of relaxed coordinates of a) C_2; b) C_4; c) C_6; d) C_8 and e) C_{10} alkene chains adsorbed on the five bilayers H−Si(111) slab (H atoms and the moiety only adsorbed over the top layer) in the most stable adsorption configurations.

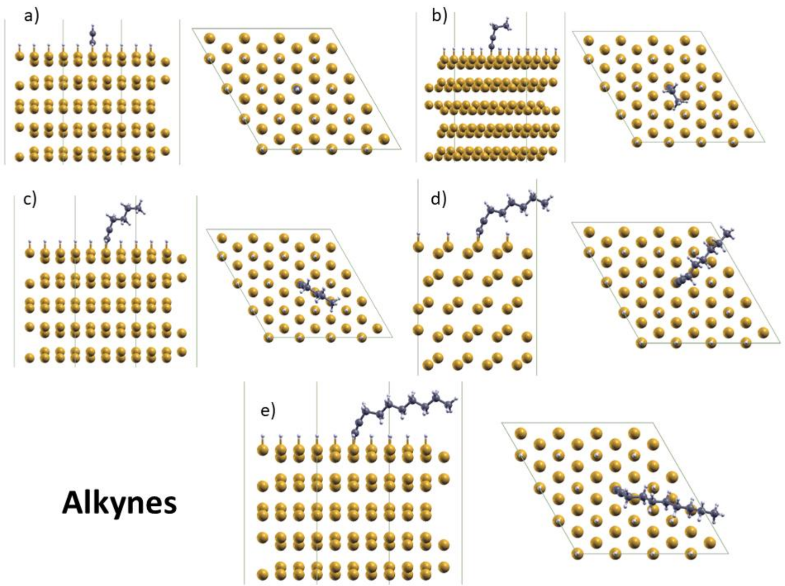

Figure S3. Side and top view of relaxed coordinates of a) C_2; b) C_4; c) C_6; d) C_8 and e) C_{10} alkyne chains adsorbed on the five bilayers H−Si(111) slab (H atoms and the moiety only adsorbed over the top layer) in the most stable adsorption configurations.

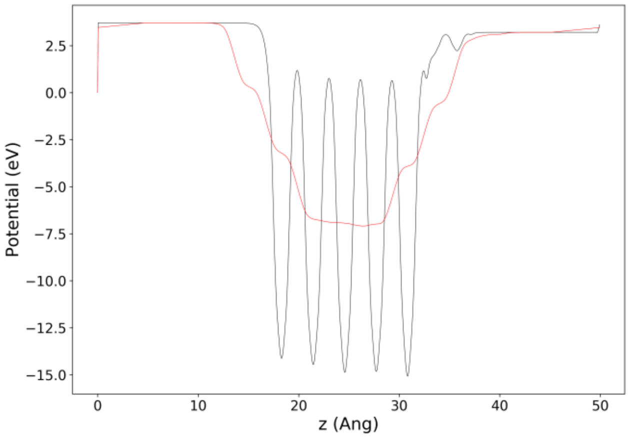

Figure S4. Plane-averaged electrostatic energy calculated on 1 (black) and 5 (red) Å step of C_2 alkyl moiety adsorbed on H−Si(111) slab. We estimated the vacuum level of each adsorption configuration as the constant plane-averaged electrostatic energy in the vacuum gap far away from the top atomic layer.

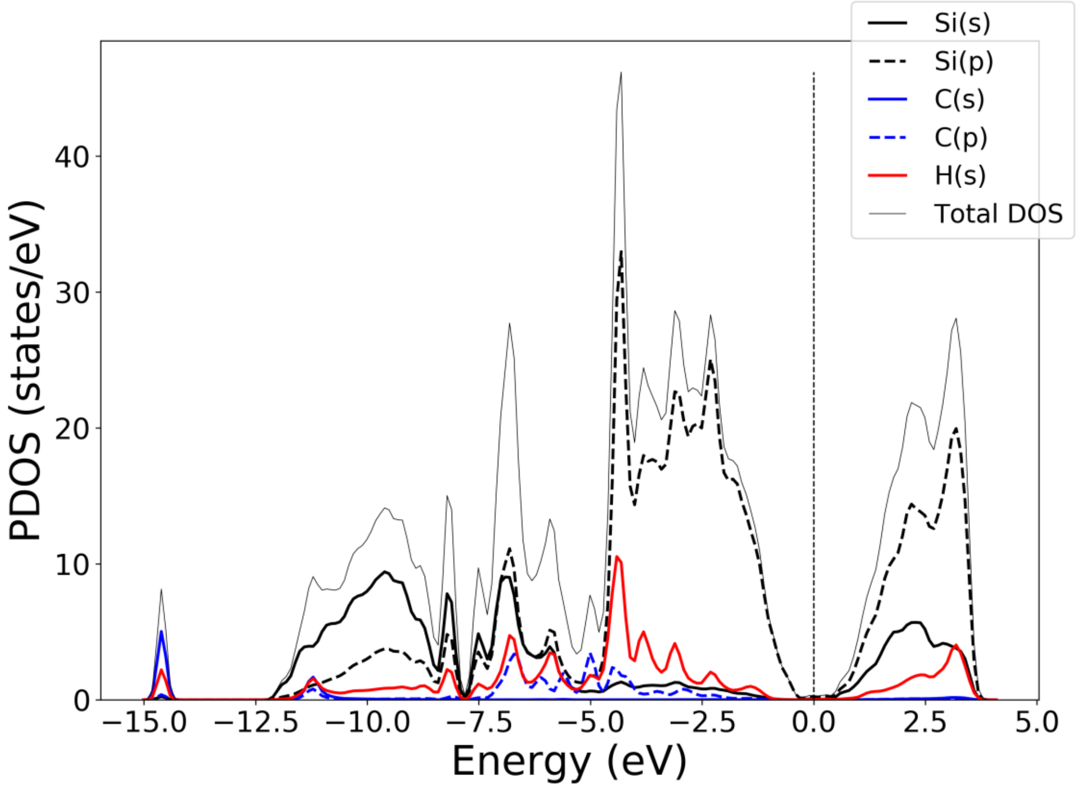

Figure S5. Projected density of states (PDOS) of C_2 alkyl moiety adsorbed on H−Si(111) slab.

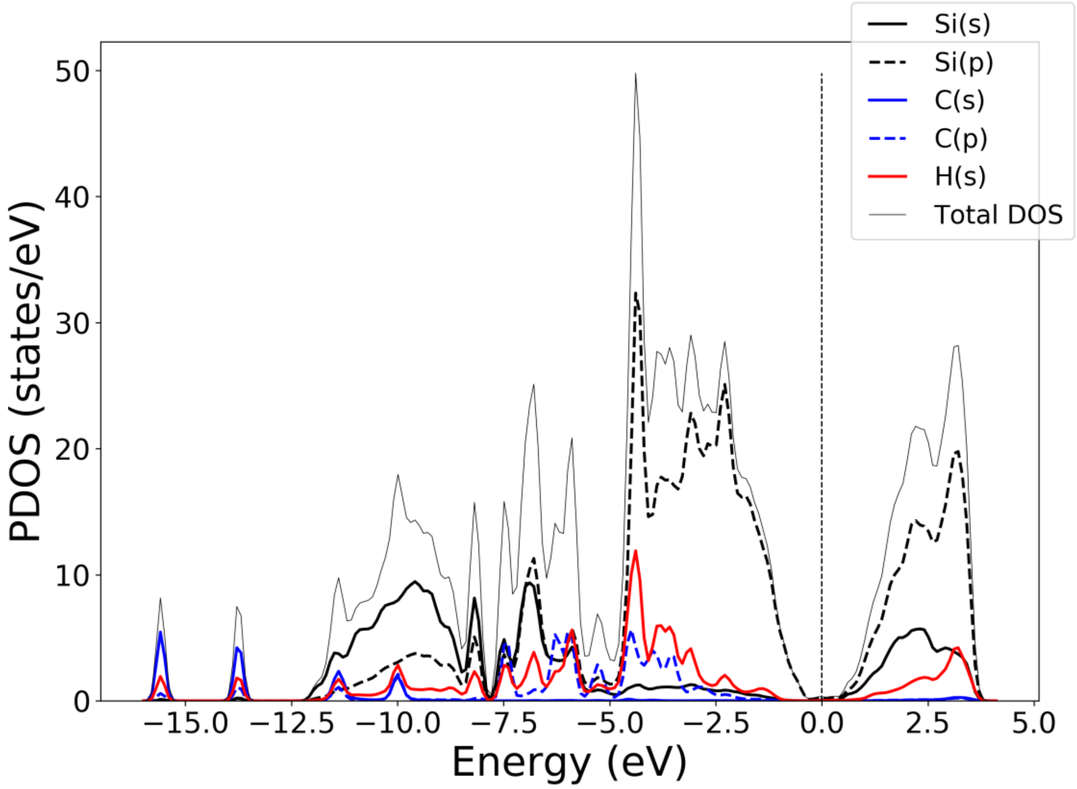

Figure S6. Projected density of states (PDOS) of C_4 alkyl moiety adsorbed on H−Si(111) slab.

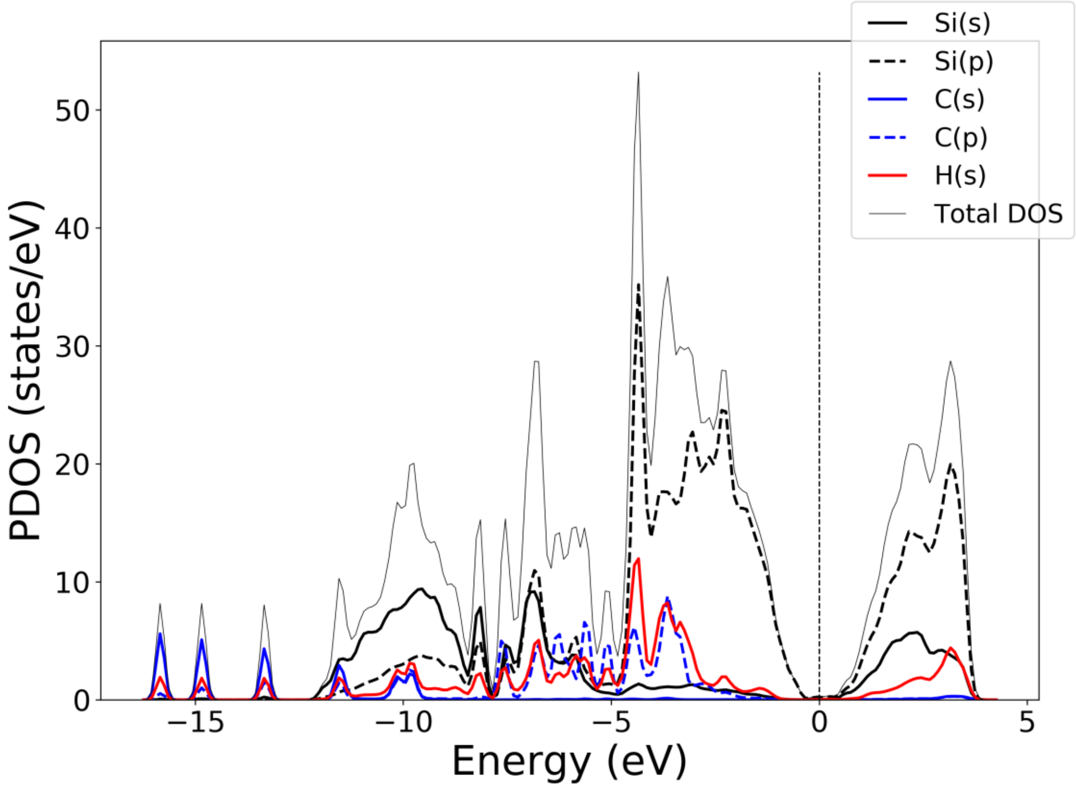

Figure S7. Projected density of states (PDOS) of C_6 alkyl moiety adsorbed on H−Si(111) slab.

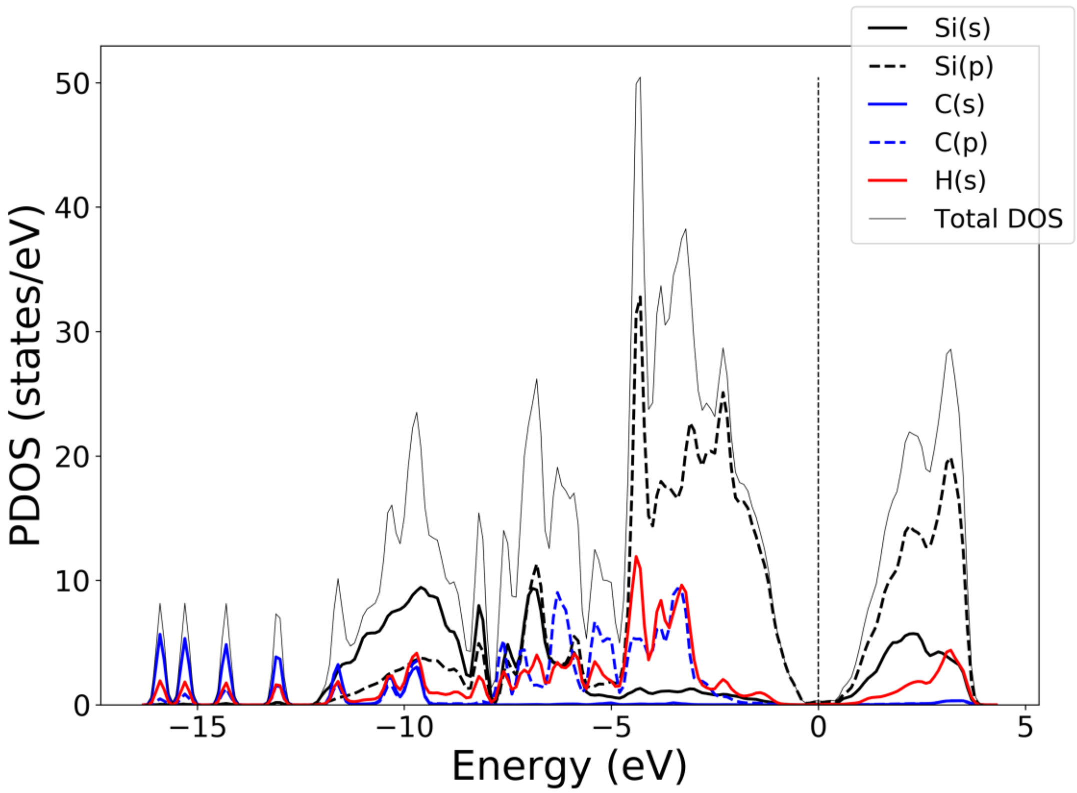

Figure S8. Projected density of states (PDOS) of C_8 alkyl moiety adsorbed on H−Si(111) slab.

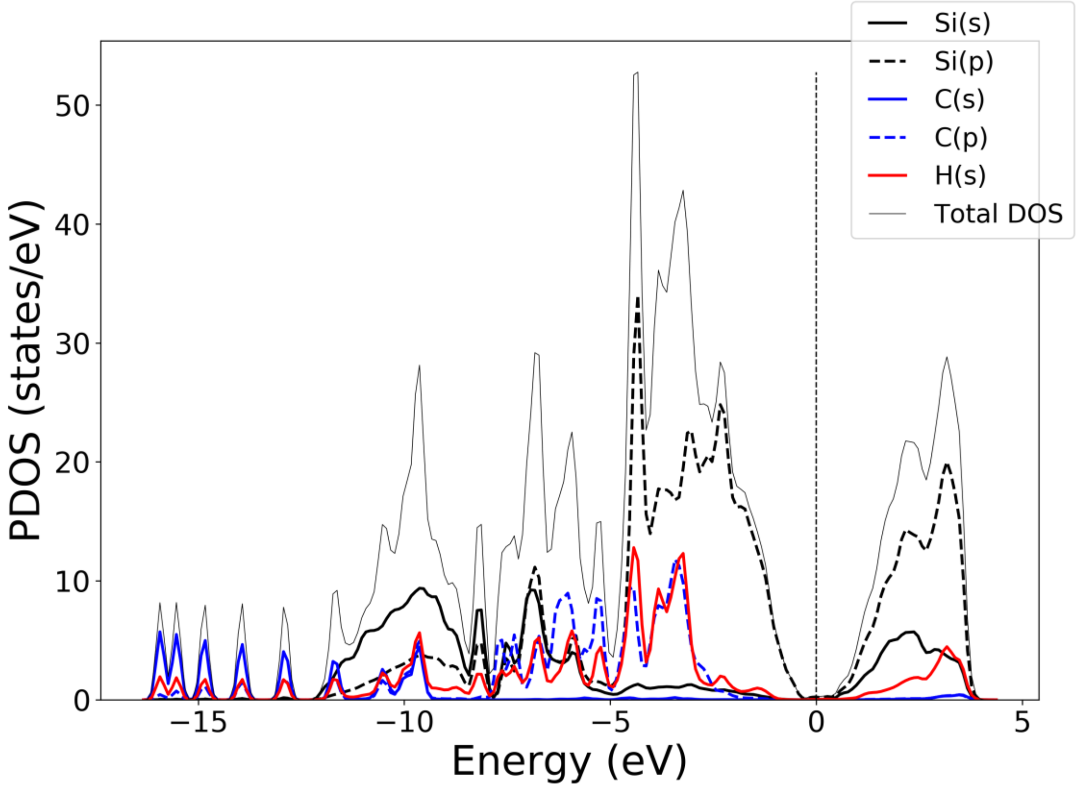

Figure S9. Projected density of states (PDOS) of C_{10} alkyl moiety adsorbed on H−Si(111) slab.

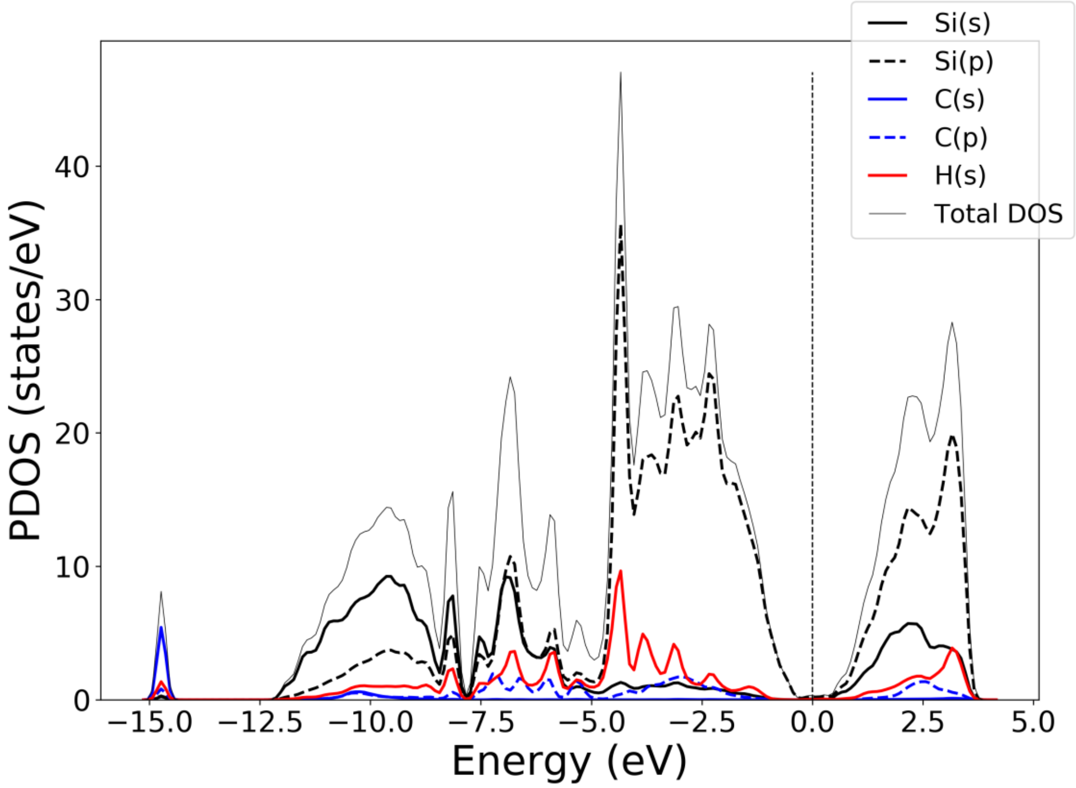

Figure S10. Projected density of states (PDOS) of C_2 alkenyl moiety adsorbed on H−Si(111) slab.

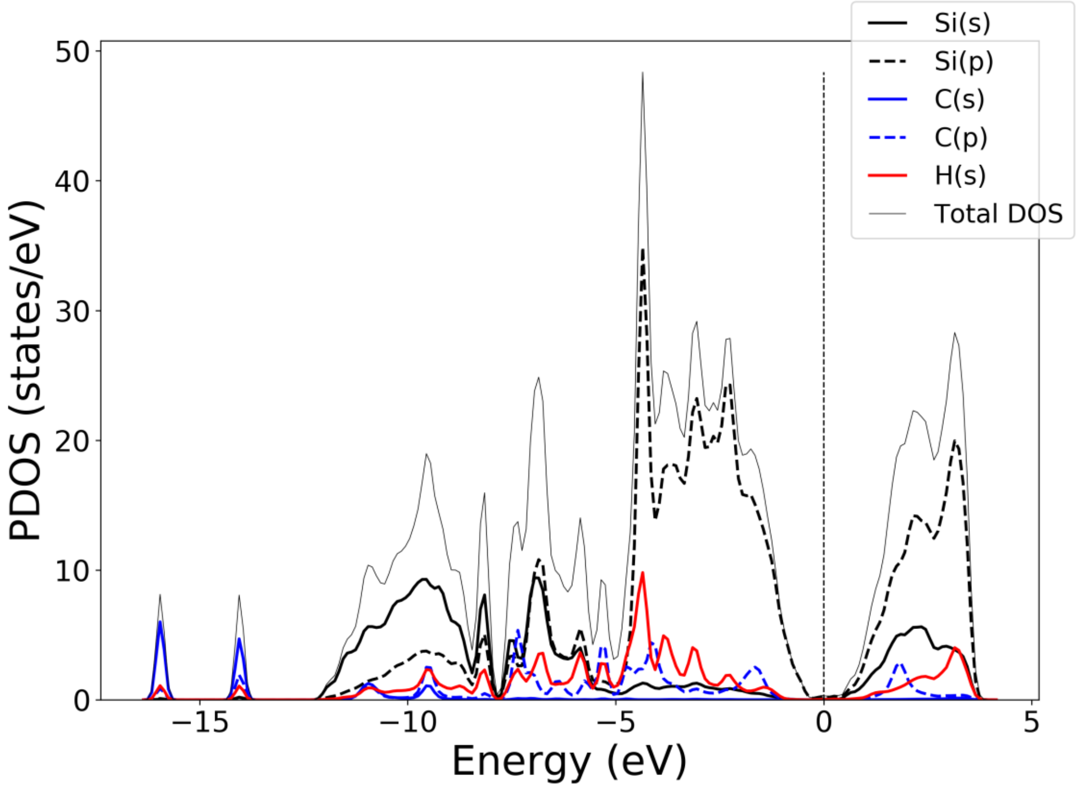

Figure S11. Projected density of states (PDOS) of C_4 alkenyl moiety adsorbed on H−Si(111) slab.

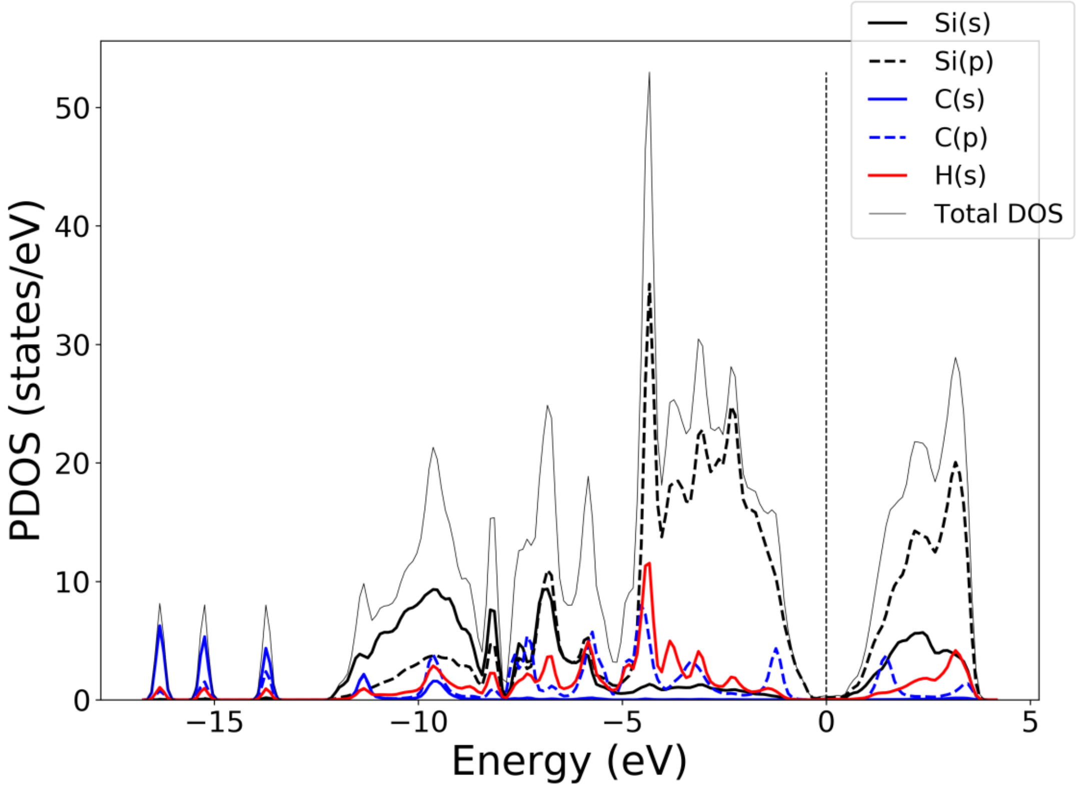

Figure S12. Projected density of states (PDOS) of C_6 alkenyl moiety adsorbed on H−Si(111) slab.

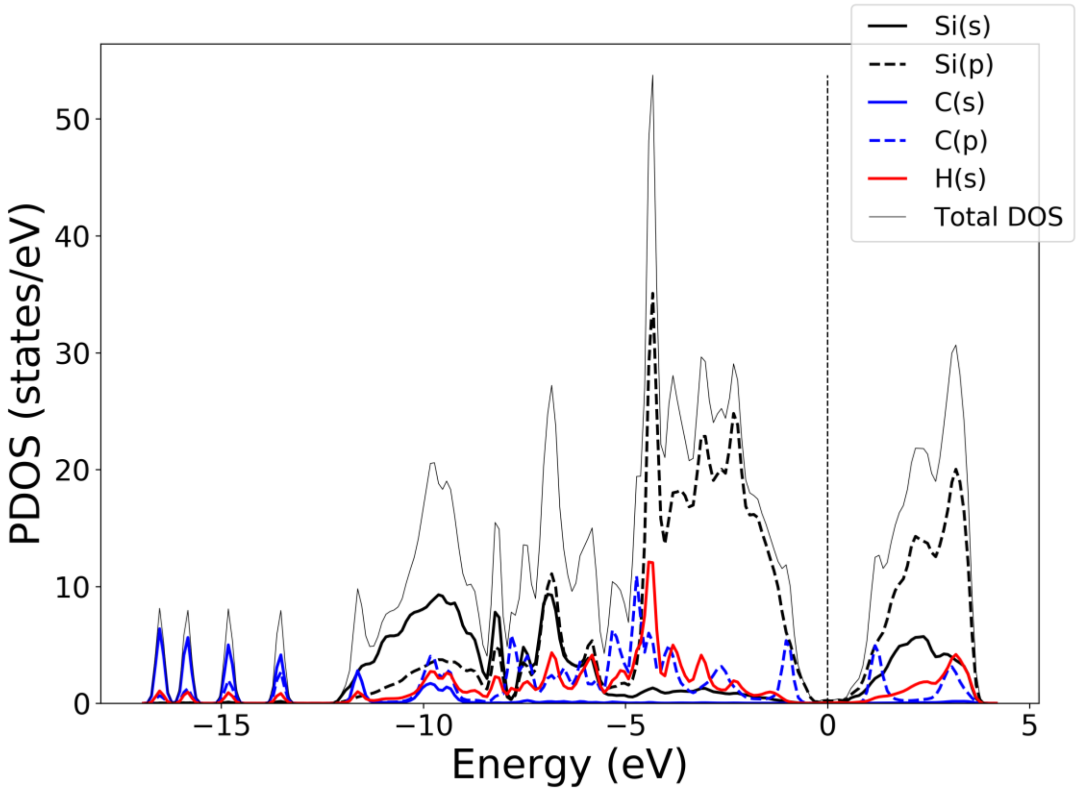

Figure S13. Projected density of states (PDOS) of C_8 alkenyl moiety adsorbed on H−Si(111) slab.

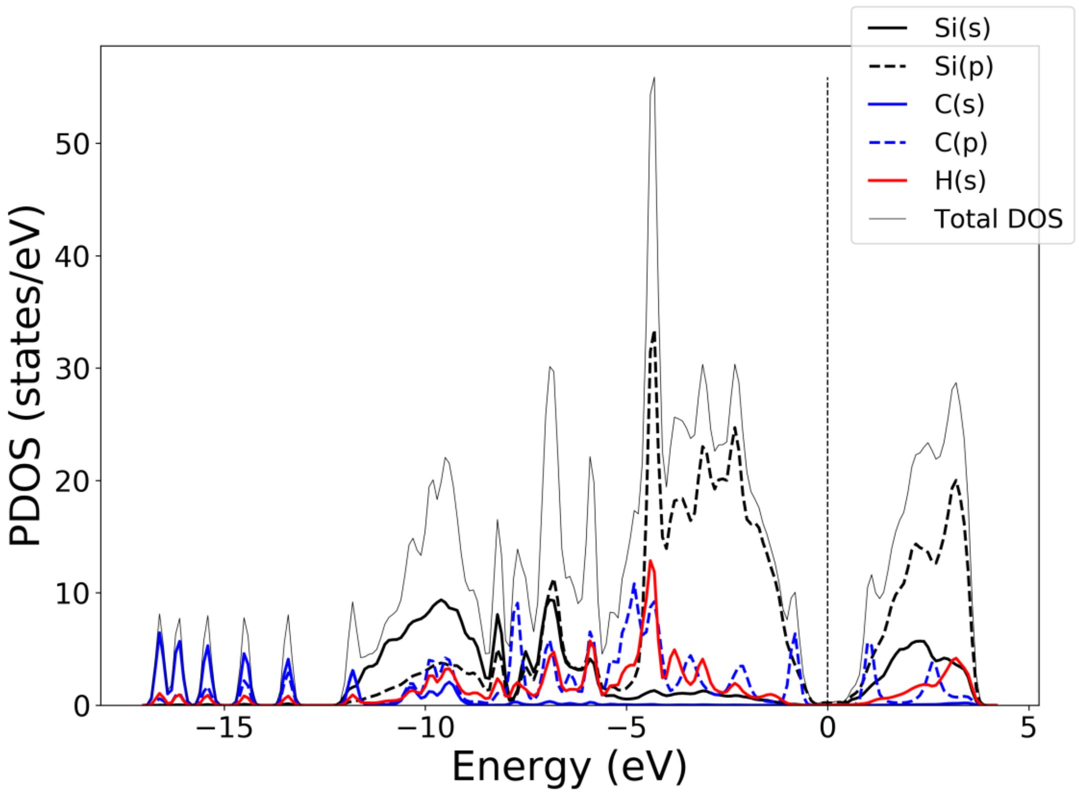

Figure S14. Projected density of states (PDOS) of C_{10} alkenyl moiety adsorbed on H−Si(111) slab.

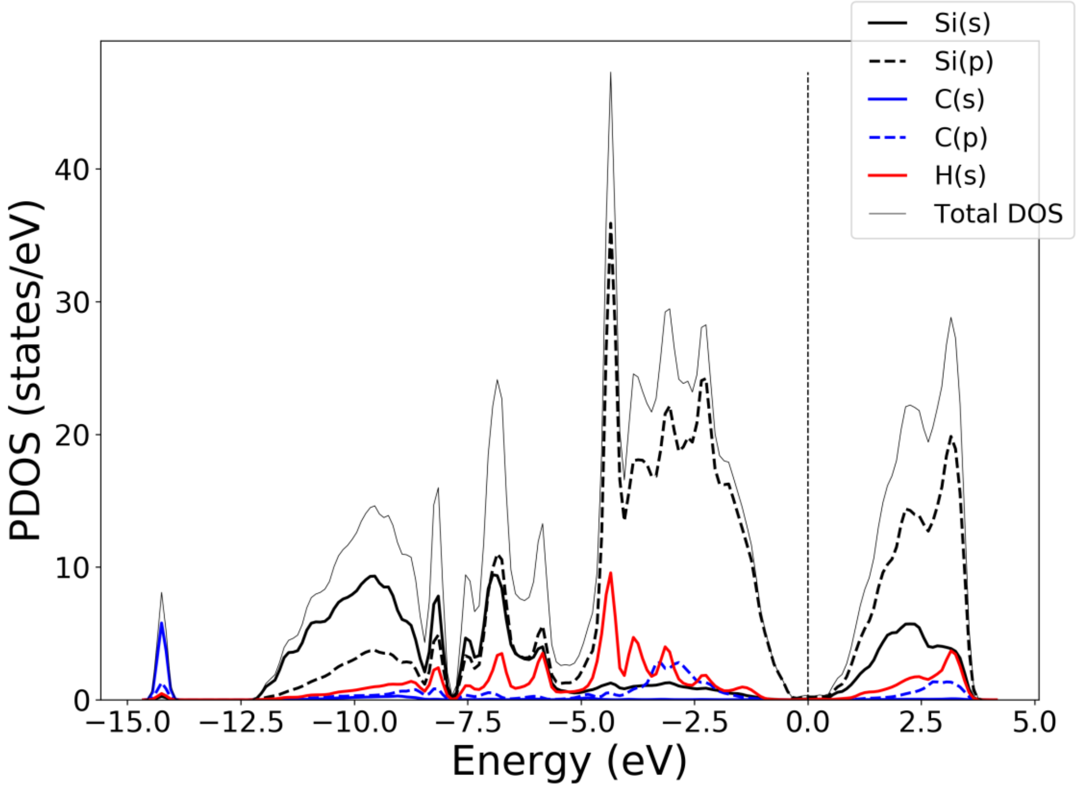

Figure S15. Projected density of states (PDOS) of C_2 alkynyl moiety adsorbed on H−Si(111) slab.

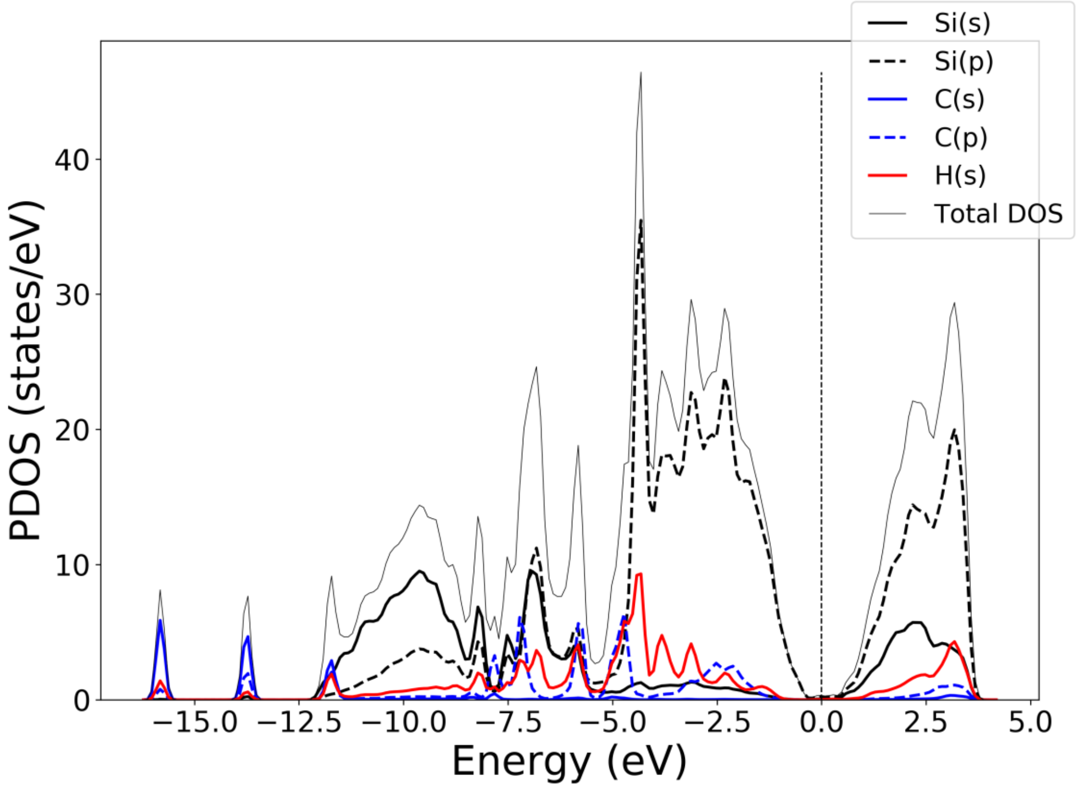

Figure S16. Projected density of states (PDOS) of C_4 alkynyl moiety adsorbed on H−Si(111) slab.

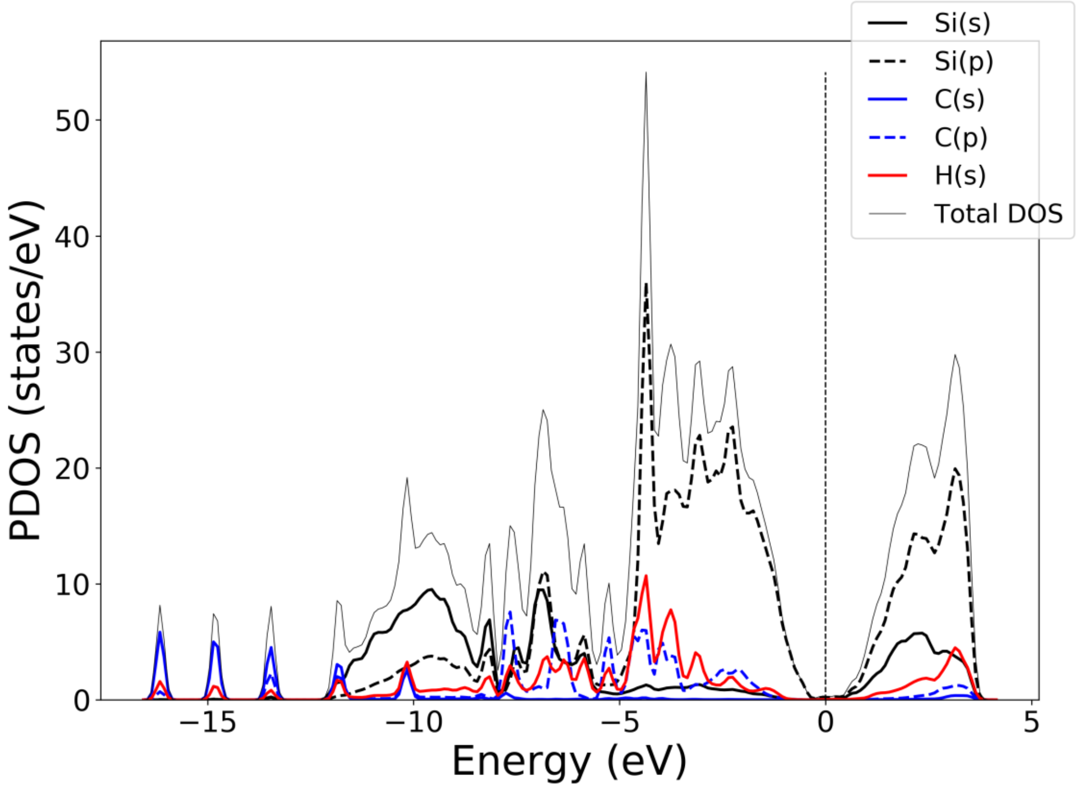

Figure S17. Projected density of states (PDOS) of C_6 alkynyl moiety adsorbed on H−Si(111) slab.

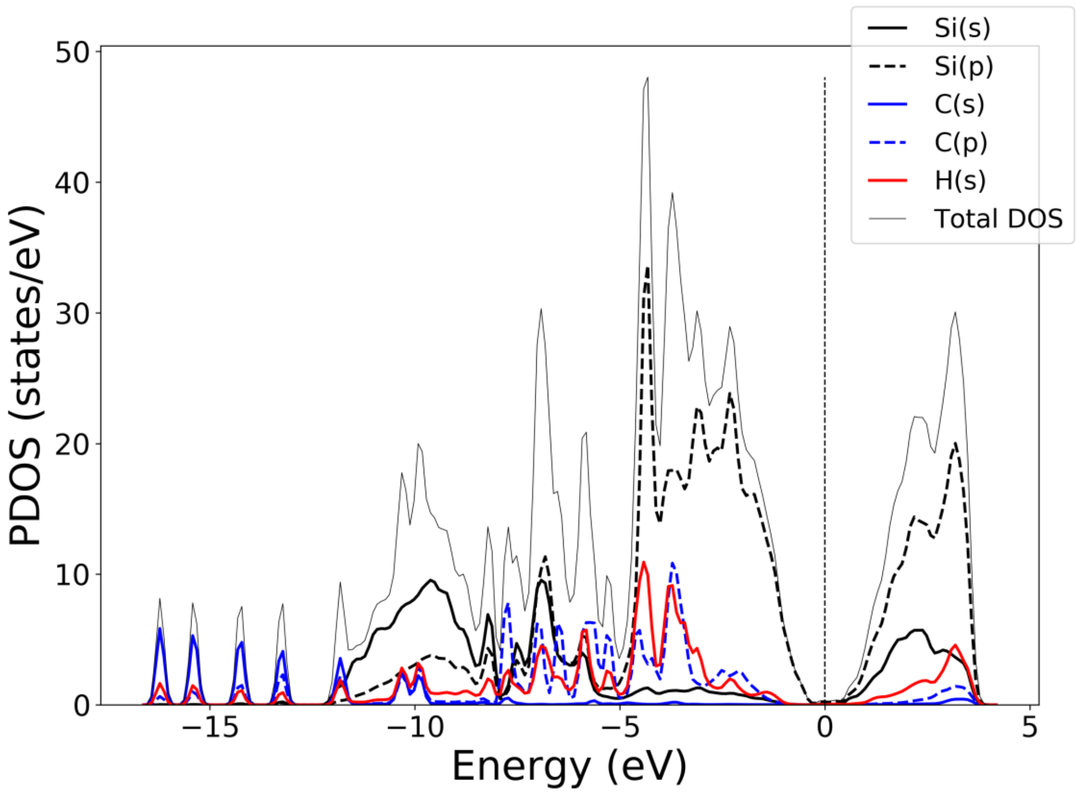

Figure S18. Projected density of states (PDOS) of C_8 alkynyl moiety adsorbed on H−Si(111) slab.

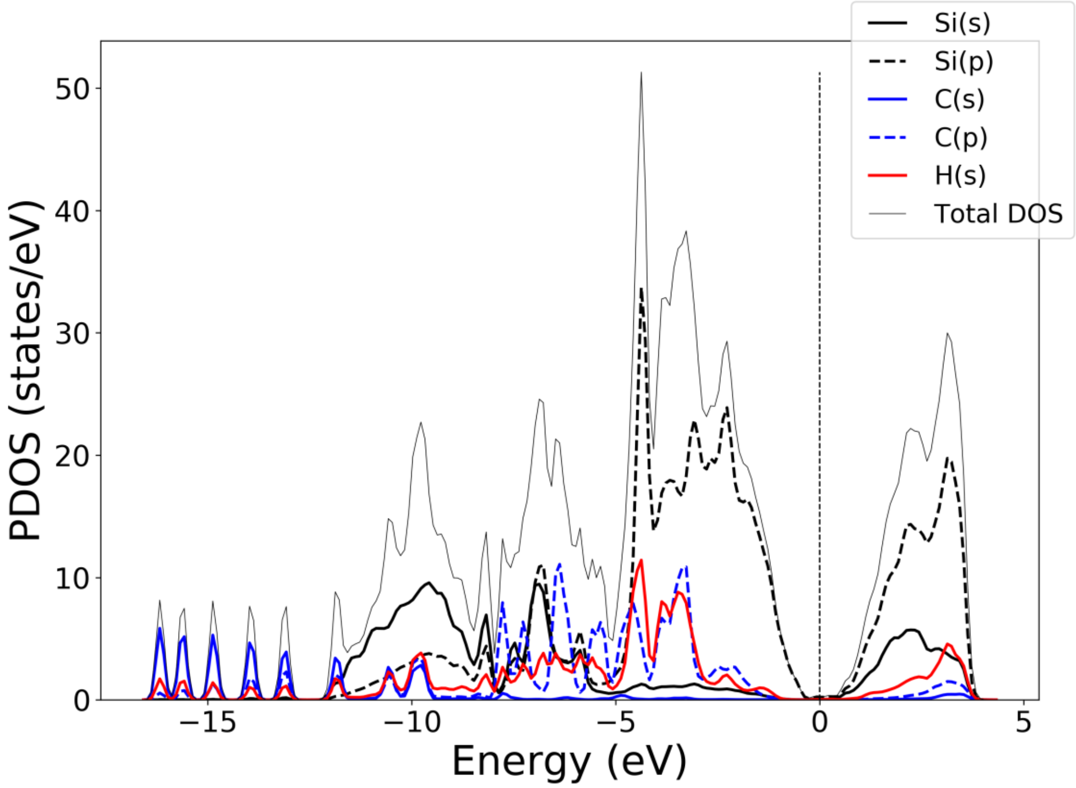

Figure S19. Projected density of states (PDOS) of C_{10} alkynyl moiety adsorbed on H−Si(111) slab.

Table S1. Ab initio energy levels of the aliphatic chains (alkyl moieties) adsorbed on the H−Si(111) surface.

**Chain**	**Valence Band Edge (eV)**	**Conduction Band Edge (eV)**	**HOMO Energy (eV)**	**LUMO Energy (eV)**
C_2	-4.923	-4.336	-5.756	-1.256
C_4	-4.928	-4.342	-5.731	-1.131
C_6	-4.934	-4.347	-5.722	-1.072
C_8	-4.881	-4.294	-5.716	-1.066
C_{10}	-4.907	-4.320	-5.712	-1.012
Si slab full H	-5.054	-4.467	–	–

Table S2. Ab initio energy levels of the aliphatic chains (1-alkenyl moieties) adsorbed on the H−Si(111) surface.

**Chain**	**Valence Band Edge (eV)**	**Conduction Band Edge (eV)**	**HOMO Energy (eV)**	**LUMO Energy (eV)**
C_2	-4.961	-4.375	-6.586	-1.786
C_4	-4.879	-4.292	-5.926	-2.456
C_6	-4.844	-4.257	-5.490	-2.770
C_8	-4.797	-4.211	-5.211	-3.001
C_{10}	-4.748	-4.162	-4.955	-3.155

Table S3. Ab initio energy levels of the aliphatic chains (1-alkynyl moieties) adsorbed on the H−Si(111) surface.

**Chain**	**Valence Band Edge (eV)**	**Conduction Band Edge (eV)**	**HOMO Energy (eV)**	**LUMO Energy (eV)**
C_2	-4.945	-4.358	-6.611	-1.611
C_4	-4.759	-4.172	-6.296	-1.296
C_6	-4.783	-4.197	-6.347	-1.327
C_8	-4.822	-4.236	-6.329	-1.309
C_{10}	-4.785	-4.198	-6.342	-1.372
